# Perioperative immunotherapy for stage II-III non-small cell lung cancer: a meta-analysis base on randomized controlled trials

**DOI:** 10.3389/fonc.2024.1351359

**Published:** 2024-02-22

**Authors:** Anping Yu, Feng Fu, Xiongying Li, Mengxin Wu, Meijian Yu, Wenxiong Zhang

**Affiliations:** ^1^ Department of Oncology, Fengcheng People’s Hospital, Yichun, China; ^2^ Department of Oncology, The Affiliated Fengcheng Hospital of Yichun University, Yichun, China; ^3^ Department of Oncology, Shangrao People’s Hospital, Shangrao, China; ^4^ Department of Thoracic Surgery, The Second Affiliated Hospital of Nanchang University, Nanchang, China

**Keywords:** immunotherapy, neoadjuvant, adjuvant, surgery, non-small cell lung cancer, meta-analysis

## Abstract

**Background:**

In recent years, we have observed the pivotal role of immunotherapy in improving survival for patients with non-small cell lung cancer (NSCLC). However, the effectiveness of immunotherapy in the perioperative (neoadjuvant + adjuvant) treatment of resectable NSCLC remains uncertain. We conducted a comprehensive analysis of its antitumor efficacy and adverse effects (AEs) by pooling data from the KEYNOTE-671, NADIM II, and AEGEAN clinical trials.

**Methods:**

For eligible studies, we searched seven databases. The randomized controlled trials (RCTs) pertaining to the comparative analysis of combination neoadjuvant platinum-based chemotherapy plus perioperative immunotherapy (PIO) versus perioperative placebo (PP) were included. Primary endpoints were overall survival (OS) and event-free survival (EFS). Secondary endpoints encompassed drug responses, AEs, and surgical outcomes.

**Results:**

Three RCTs (KEYNOTE-671, NADIM II, and AEGEAN) were included in the final analysis. PIO group (neoadjuvant platinum-based chemotherapy plus perioperative immunotherapy) exhibited superior efficacy in OS (hazard ratio [HR]: 0.63 [0.49-0.81]), EFS (HR: 0.61 [0.52, 0.72]), objective response rate (risk ratio [RR]: 2.21 [1.91, 2.54]), pathological complete response (RR: 4.36 [3.04, 6.25]), major pathological response (RR: 2.79 [2.25, 3.46]), R0 resection rate (RR: 1.13 [1.00, 1.26]) and rate of adjuvant treatment (RR: 1.08 [1.01, 1.15]) compared with PP group (neoadjuvant platinum-based chemotherapy plus perioperative placebo). In the subgroup analysis, EFS tended to favor the PIO group in almost all subgroups. BMI (>25), T stage (IV), N stage (N1-N2) and pathological response (with pathological complete response) were favorable factors in the PIO group. In the safety assessment, the PIO group exhibited higher rates of serious AEs (28.96% vs. 23.51%) and AEs leading to treatment discontinuation (12.84% vs. 5.81%). Meanwhile, although total adverse events, grade 3-5 adverse events, and fatal adverse events tended to favor the PP group, the differences were not statistically significant.

**Conclusion:**

PIO appears to be superior to PP for resectable stage II-III NSCLC, demonstrating enhanced survival and pathological responses. However, its elevated adverse event (AE) rate warrants careful consideration.

**Systematic review registration:**

https://www.crd.york.ac.uk/PROSPERO/#recordDetails, identifier CRD42023487475.

## Introduction

For decades, lung cancer (LC) has been the leading global cause of cancer-related deaths, with over 80% attributed to non-small cell lung cancer (NSCLC) (1, 2). Comprehensive treatment based on surgery is the standard of care for selected resectable stages II-III NSCLC (3). In previous approaches to neoadjuvant and adjuvant treatment for stage II-III NSCLC, chemotherapy played a vital role, but its solitary use yielded unsatisfactory results (4). In recent years, immunotherapy has gained widespread acceptance in solid tumor treatment, demonstrating superior efficacy in both neoadjuvant and adjuvant treatment for resectable NSCLC (5–7). Nevertheless, controversy persists in clinical settings regarding whether perioperative immunotherapy (neoadjuvant+adjuvant) can yield superior results (8).

The use of immunotherapy in the perioperative period of resectable lung cancer has been a hot topic in recent years. In neoadjuvant therapy, the CheckMate 816 study demonstrated that the addition of nivolumab to platinum-based chemotherapy (PBC) could significantly increase event-free survival (EFS) and drug responses (9). Similar results were also validated in the TD-FOREKNOW study (Camrelizumab) (10). In adjuvant therapy, the KEYNOTE-091 study showed that the addition of pembrolizumab to PBC could significantly increase disease-free survival (DFS) (11). The IMpower010 study also confirmed that adding atezolizumab to PBC could improve DFS and overall survival (OS), especially in patients with programmed cell death 1 ligand 1 (PD-L1)-positive NSCLC (12). Regarding the use of immunotherapy in combination of neoadjuvant and adjuvant therapy, both the KEYNOTE-671 study (pembrolizumab) and the AEGEAN study (durvalumab) found that perioperative immunotherapy could significantly improve OS and EFS, and similar results were also validated in the NADIM II study (nivolumab) (13–15).

This study conducted a meta-analysis based on randomized controlled trials (RCTs) to evaluate the impact of perioperative immunotherapy with neoadjuvant PBC on survival, pathological responses, and adverse reactions.

## Materials and methods

This study was conducted in accordance with PRISMA guidelines and registered in PROSPERO (ID: CRD42023487475) (**Supplementary Table S1**).

### Search strategy

The search strategy involved the use of keywords: “lung cancer,” “randomized,” and immune checkpoint inhibitors (nivolumab, pembrolizumab, treprinumab, cedilimumab, camrelizumab, tislelizumab, penpulimab, zimberelimab, serplulimab, durvalumab, atezolizumab, envolizumab, sugemalimab, adebrelimab, ipilimumab, and tremelimumab). Seven databases (PubMed, ScienceDirect, Ovid MEDLINE, the Cochrane Library, Scopus, EMBASE and Web of Science) were thoroughly searched for eligible RCTs from the inception of the databases to November 15, 2023 (**Supplementary Table S2**). Additionally, we reviewed the reference lists of the included RCTs to identify any further eligible studies.

### Selection criteria

The studies published in English were selected following PICOS criteria:

(1) Participants (P): patients with stage II-III NSCLC, evaluated per the American Joint Committee on Cancer staging system, 8th edition (16).
(2) Intervention (I): neoadjuvant (PBC+immunotherapy) + adjuvant (immunotherapy), defined as the perioperative immunotherapy (PIO) group.
(3) Control (C): neoadjuvant (PBC+placebo) + adjuvant (placebo), defined as the perioperative placebo (PP) group.
(4) Outcomes (O): survival (OS, EFS), pathological responses, and adverse events (AEs).
(5) Study design (S): RCTs.

Articles lacking initial data, as well as meta-analyses, conference articles, and case reports, were not considered for inclusion. Distinct articles covering the same trial with diverse outcomes were included, but for identical outcomes, only the most recent data were utilized in the analysis.

### Data extraction

Two investigators independently extracted data, including study characteristics (publication date, first author, etc.), participant details (sex, age, etc.), cancer specifics (histopathology, stage, etc.), antitumor effectiveness (OS, EFS, pathological responses, etc.), and counts of adverse events (total AEs, serious AEs, etc.). Disagreements were resolved through a process of re-evaluation and discussion.

### Outcome assessments

The primary endpoints analyzed were OS and EFS. Simultaneously, the overall survival rate (OSR) and event-free survival rate (EFSR) at 6, 12, 18, 24, 30, 36, 42, and 48 months were compared between the two groups. Additionally, we examined EFS within specific subgroups, including patient characteristics (sex, age, etc.), histologic features, pathological stage, T stage, N stage, PD-L1 tumor cell proportion score (TPS), epidermal growth factor receptor (EGFR) mutation, anaplastic lymphoma kinase (ALK) translocation, pathological response (major pathological response [MPR]), and pathological response (pathological complete response [PCR]).

### Quality assessment

We assessed the quality of RCTs using the Jadad scale, a 5-point system reflecting randomization, blinding, and patient inclusion. A score of ≥3 points was considered indicative of high quality (17). Additionally, the Cochrane Risk Assessment Tool was employed, which evaluates bias related to selection, performance, detection, attrition, and reporting and categorizes risk as low, unclear, or high (18). The results are presented in a bias graph.

We assessed the quality of the results using the Grading of Recommendations, Assessment, Development, and Evaluation (GRADE) method, which primarily encompasses bias, indirectness, inaccuracy, and publication bias. The outcomes are classified into four levels: very low, low, medium, and high (19).

### Statistical analysis

The pooled data were assessed using Review Manager 5.3. Hazard ratios (HR) were employed for the analysis of survival data, favoring the PIO group when HR < 1. For dichotomous variables, we used the risk ratio (RR), with results favoring the PP group when RR > 1, particularly in the AE analysis. Conversely, support for the PIO group emerged in the analysis of OSR, EFSR, and drug responses. Heterogeneity was assessed using the *I^2^* statistic and χ2 test. In cases where *I^2^* was less than 50% or p was greater than 0.1, indicating the absence of significant heterogeneity, we employed a fixed-effects model; otherwise, a random-effects model was utilized. Statistical significance was defined by P values less than 0.05, and we assessed publication bias by visually inspecting funnel plots.

## Results

### Search results

**Figure 1:**
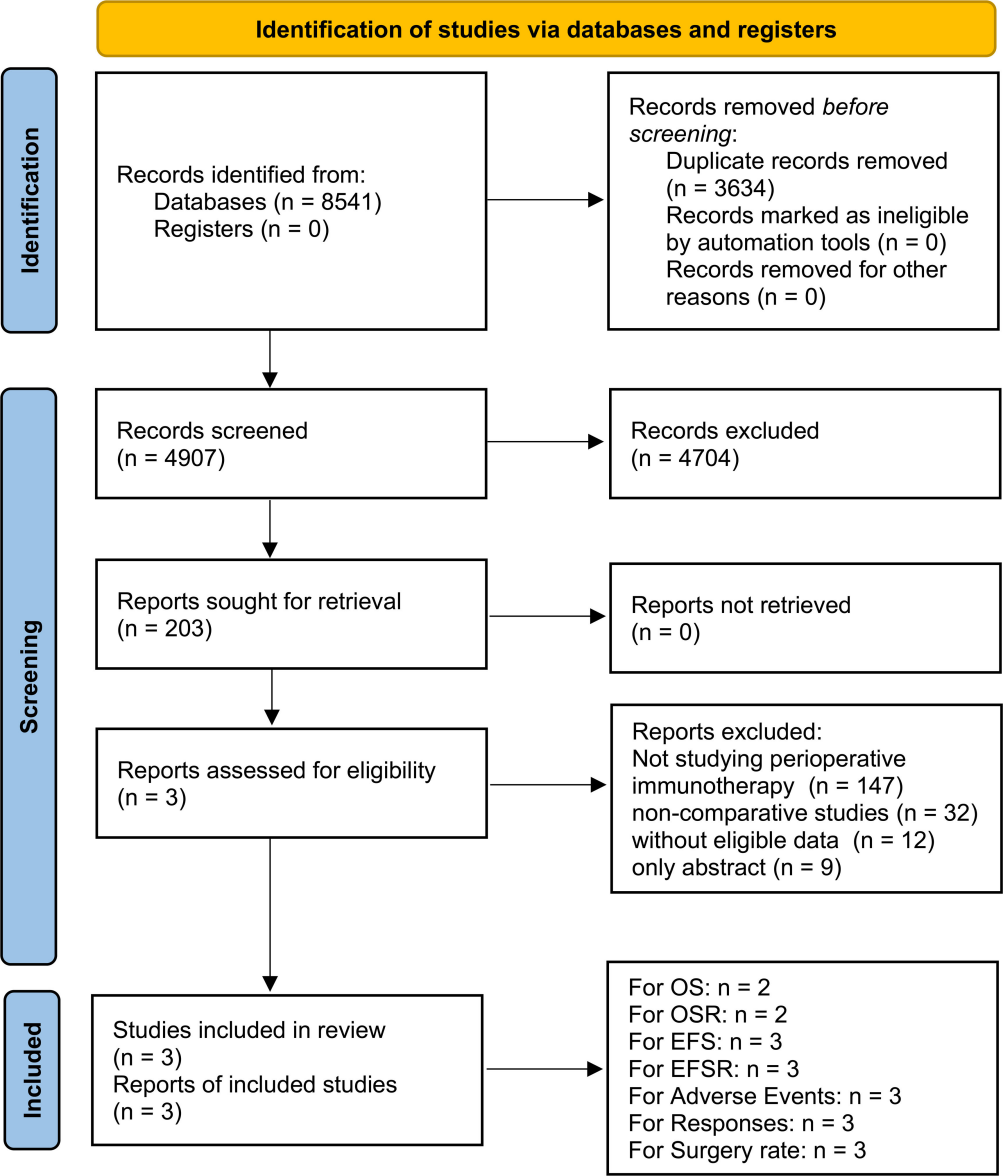
Study selection flow.

Three high-quality RCTs (KEYNOTE-671, NADIM II, and AEGEAN) were included in the analysis. The PIO group included 820 patients, and the PP group included 803 patients (**Figure 1**, **Supplementary Figure S1**, **Supplementary Table S3**) (13–15). These comprised two global multicenter studies (KEYNOTE-671 and AEGEAN) and one study conducted in Spain (NADIM II) (13–15). As per the GRADE method, the quality of all results was categorized within the medium-high range (**Supplementary Table S4**). **Table 1** provided a summary of the baseline information for the included studies.

**Table 1:** Characteristics of the three randomized controlled trials (KEYNOTE-671, NADIM II and AEGEAN).

Study	KEYNOTE-671		NADIM II		AEGEAN	
**Register number**	NCT03425643		NCT03838159		NCT03800134	
**Design**	RCT		RCT		RCT	
**Clinical trial stage**	Phase III		Phase II		Phase III	
**Included articles**	Wakelee 2023 (13)		Provencio 2023 (14)		Heymach 2023 (15)	
**Country**	Global multicenter		Spain		Global multicenter	
**Period**	2018.04-2021.12		2019.06-2021.02		2019.01-2022.04	
**Treatment arm**	PIO	PP	PIO	PP	PIO	PP
**Neoadjuvant therapy**	PBC+Pembro 4 cycles	PBC+Placebo 4 cycles	PBC+Nivo 3 cycles	PBC+Placebo 3 cycles	PBC+Durva 4 cycles	PBC+Placebo 4 cycles
**Adjuvant therapy**	Pembro up to 13 cycles	Placebo up to 13 cycles	Nivo up to 6 cycles	Placebo up to 6 cycles	Durva up to 12 cycles	Placebo up to 12 cycles
**Patients (n)**	397	400	57	29	366	374
**Sex (M/F)**	279/118	284/116	36/21	16/13	252/114	278/96
**Median age (year)**	63	64	65	63	65	65
Race category						
White	250	239	57	29	206	191
Asian	124	125	0	0	143	164
Others	23	36	0	0	17	19
ECOG status						
0	253	246	31	16	251	255
1	144	154	26	13	115	119
Smoking status						
Current	96	103	30	21	95	95
Former	247	250	22	8	220	223
Never	54	47	5	0	51	56
Histologic classification						
Squamous	226	173	21	14	169	193
Nonsquamous	171	227	36	15	197	181
TNM stage						
II	118	121	0	0	104	110
IIIA	217	225	44	24	174	165
IIIB	62	54	13	5	88	98
PD-L1 expression						
<1%	138	151	20	9	122	125
1-49%	127	115	21	11	135	142
>50%	132	134	16	9	109	107
**Cut off time (months)**	25.2		26.1		34	
**Tumor response assessment**	RECIST, version 1.1		RECIST, version 1.1		RECIST, version 1.1	
**Adverse events assessment**	NCI-CTCAE, version 4.03		NCI-CTCAE, version 5.0		NCI-CTCAE, version 5.0	
**Funding**	Merck Sharp and Dohme		Bristol Myers Squibb		AstraZeneca	

Durva, Durvalumab; ECOG, Eastern Cooperative Oncology Group; M/F, male/female; NCI-CTCAE, National Cancer Institute Common Terminology Criteria for Adverse; Nivo, Nivolumab; PD-L1, Programmed cell death 1 ligand 1; Pembro, Pembrolizumab; PIO, Perioperative immunotherapy; PP, Perioperative placebo; RCT, Randomized controlled trial; RECIST, Response Evaluation Criteria in Solid Tumors.

### Antitumor efficacy

**Figure 2:**
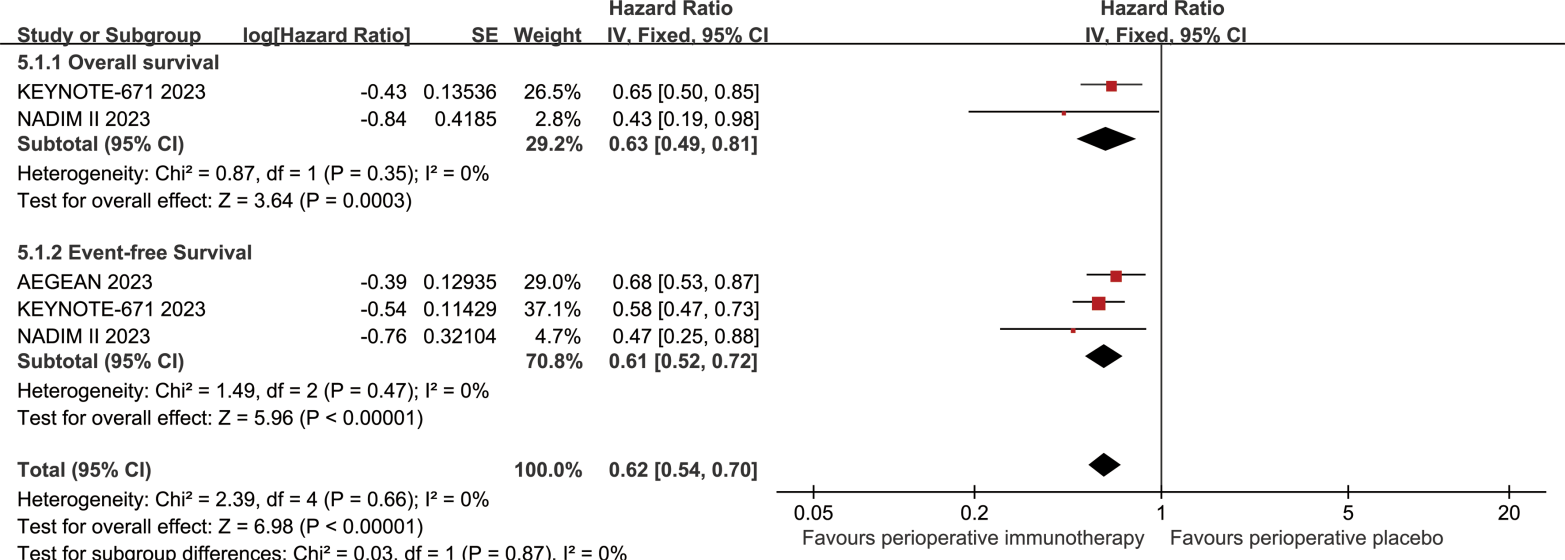
Forest plots of overall survival and event-free survival associated with perioperative immunotherapy versus perioperative placebo.

**Figure 3:**
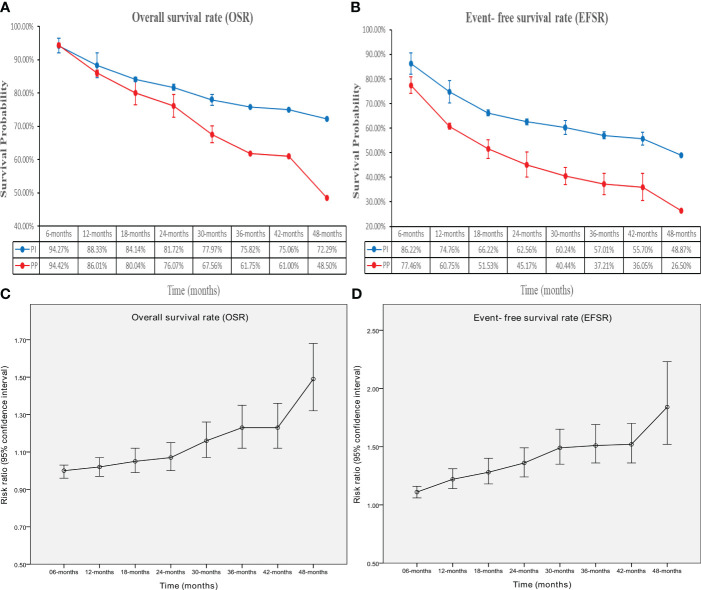
Comparisons of overall survival rate (6-48 months, **A**: trend of overall survival rate; **C**: trend of risk ratios) and event-free survival rate (6-48 months, **B**: trend of event-free survival rate; **D**: trend of risk ratios) associated with perioperative immunotherapy versus perioperative placebo according to survival time.

The OS in the PIO group surpassed that in the PP group (HR: 0.63 [0.49-0.81], p = 0.0003; **Figure 2**). At 24-48 months, OSR favored the PIO group (OSR-24 m, RR: 1.07 [1.00, 1.15]; OSR-30 m, RR: 1.16 [1.07, 1.26]; OSR-36 m, RR: 1.23 [1.12, 1.35]; OSR-42 m, RR: 1.23 [1.12, 1.36]; OSR-48 m, RR: 1.49 [1.32, 1.68]) (**Supplementary Figure S2**). As survival extended, PIO demonstrated an increasing OS advantage compared to PP (**Figures 3A, C**).

The EFS in the PIO group surpassed that in the PP group (HR: 0.61 [0.52, 0.72], p < 0.00001; **Figure 2**). At 6-48 months, EFSR favored the PIO group (EFSR-6 m, RR: 1.11 [1.06, 1.16]; EFSR-12 m, RR: 1.22 [1.14, 1.31]; EFSR-18 m, RR: 1.28 [1.18, 1.40]; EFSR-24 m, RR: 1.36 [1.24, 1.49]; EFSR-30 m, RR: 1.49 [1.35, 1.65]; EFSR-36 m, RR: 1.51 [1.36, 1.69]; EFSR-42 m, RR: 1.52 [1.36, 1.70]; EFSR-48 m, RR: 1.84 [1.52, 2.23]; **Supplementary Figure S3**). Regarding extended survival, PIO demonstrated an increasing advantage in EFS compared to PP (**Figures 3B, D**).

**Figure 4:**
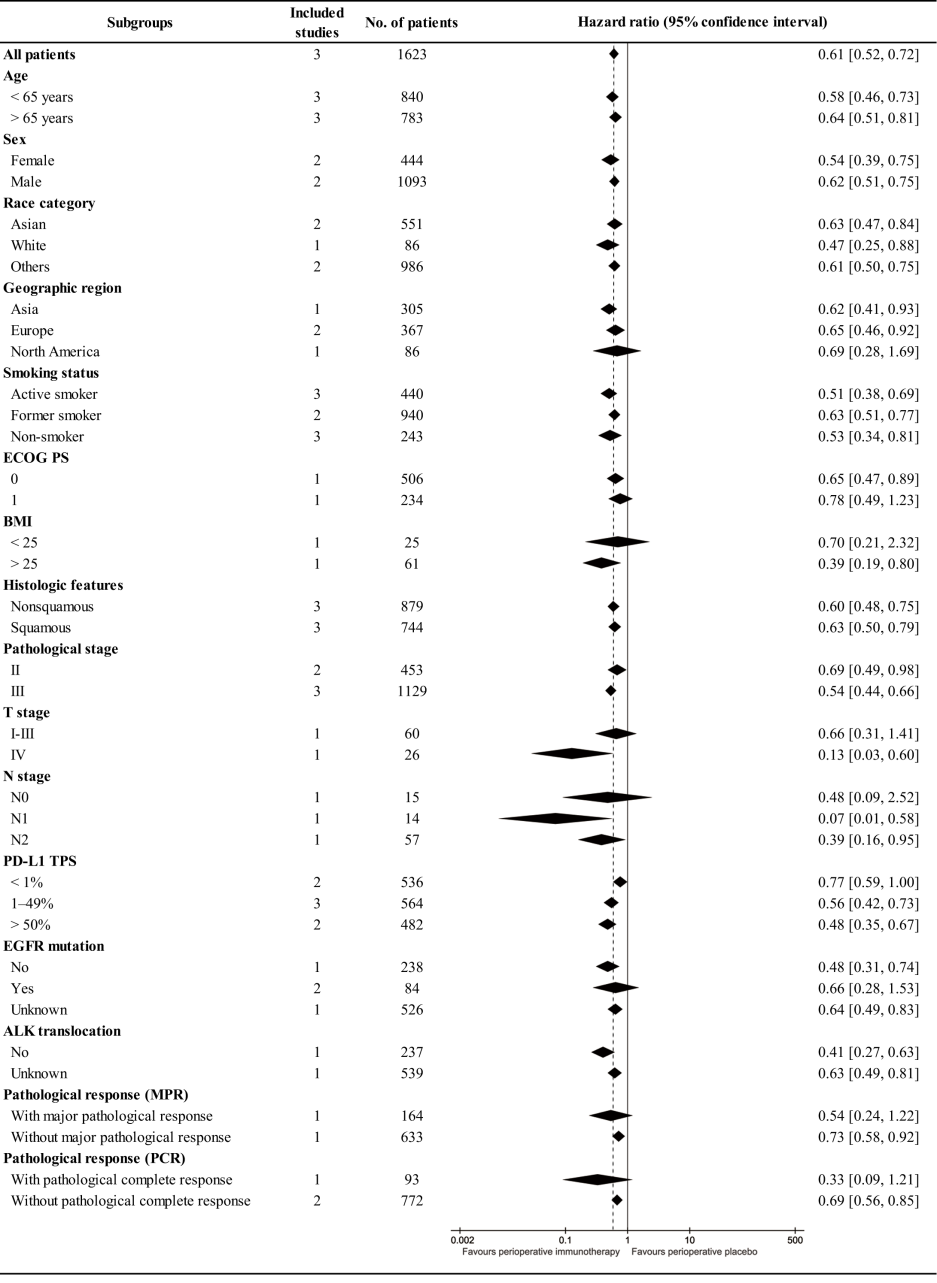
Subgroup analysis of event-free survival.

In subgroup analysis, EFS tended to favor the PIO group across most subgroups. High BMI (>25), advanced T stage (IV), involved N stage (N1-N2), and favorable pathological response (with PCR) might benefit PIO treatment. Simultaneously, the EFS advantage of PIO increased with higher PD-L1 expression (PD-L1 TPS, < 1%, RR: 0.77 [0. 59-1.00]; 1-49%, RR: 0.56 [0. 42-0.73]; > 50%, RR: 0.48 [0. 35-0.67]) (**Figure 4**).

**Figure 5:**
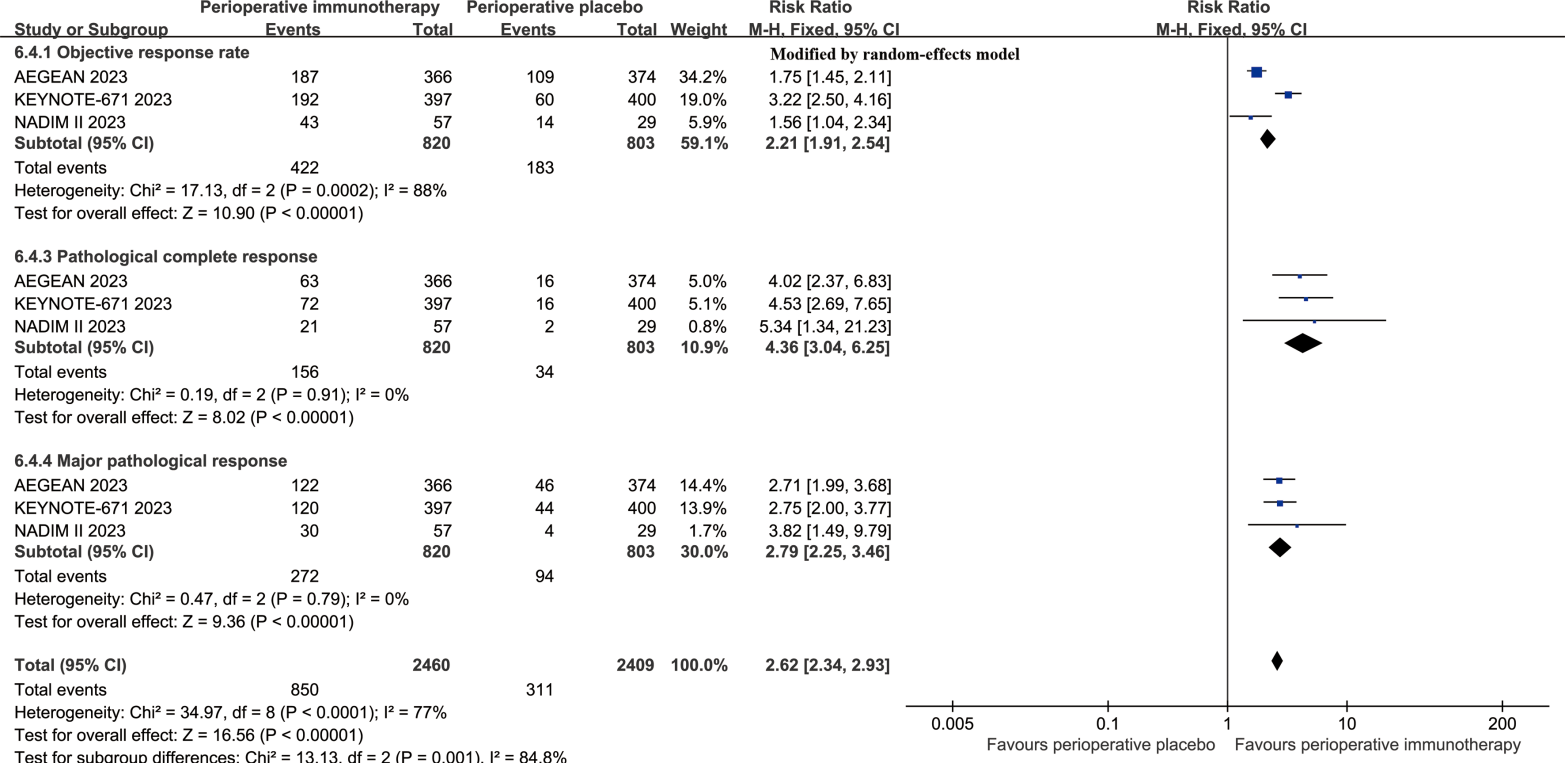
Forest plots of pathological responses (objective response rate, pathological complete response, and major pathological response) associated with perioperative immunotherapy versus perioperative placebo according to survival time.

The objective response rate (ORR, RR: 2.21 [1.91, 2.54]), PCR (RR: 4.36 [3.04, 6.25]), and MPR (RR: 2.79 [2.25, 3.46]) surpassed those in the PIO group (**Figure 5**). The surgery rates were similar between the two groups, and the R0 resection rate (RR: 1.08 [1.01, 1.16]) was higher in the PIO group (**Supplementary Figure S4**). The started rate (RR: 1.08 [1.01, 1.15]) and completed rate (RR: 1.13 [0.98, 1.30]) of adjuvant therapy tended to favor the PIO group (**Supplementary Figure S5**).

### Toxicity

To summarize, PIO treatment resulted in a greater incidence of serious AEs (28.96% vs. 23.51%, RR: 1.24 [1.05, 1.46]) and AEs leading to treatment discontinuation (ALTD, 12.84% vs. 5.81%, RR: 2.21 [1.58, 3.10]). Total AEs, grade 3-5 AEs and fatal AEs tended to favor the PP group without significant differences (**Table 2**, **Supplementary Figure S6**).

**Table 2:** Summary of adverse events.

Adverse events	Studies involved	PIO		PP		Risk ratio [95% CI]	P
		Event/total	%	Event/total	%		
During all phases							
Total adverse events	3	806/820	98.29%	781/803	97.26%	1.01 [0.99, 1.03]	0.19
Grade 3-5 adverse events	3	360/820	43.90%	324/803	40.35%	1.11 [0.99, 1.25]	0.07
Serious adverse events	2	221/763	28.96%	182/774	23.51%	1.24 [1.05, 1.46]	0.01
Fatal adverse events	2	27/763	3.54%	18/774	2.33%	1.53 [0.85, 2.74]	0.15
Adverse event leading to treatment discontinuation	2	98/763	12.84%	45/774	5.81%	2.21 [1.58, 3.10]	<0.00001
During the Neoadjuvant Treatment Phase							
Total adverse events	2	436/454	96.04%	403/429	93.94%	1.02 [0.99, 1.05]	0.23
Grade 3-5 adverse events	2	173/454	38.11%	149/429	34.73%	1.14 [0.95, 1.35]	0.15
Serious adverse events	1	56/397	14.11%	52/400	13.00%	1.09 [0.76, 1.54]	0.65
Fatal adverse events	1	3/397	0.76%	3/400	0.75%	1.01 [0.20, 4.96]	0.99
During the Surgical Treatment Phase							
Total adverse events	1	231/397	58.19%	226/400	56.50%	1.03 [0.91, 1.16]	0.63
Grade 3-5 adverse events	1	84/397	21.16%	68/400	17.00%	1.24 [0.93, 1.66]	0.14
Serious adverse events	1	59/397	14.86%	54/400	13.50%	1.10 [0.78, 1.55]	0.58
Fatal adverse events	1	9/397	2.27%	5/400	1.25%	1.81 [0.61, 5.36]	0.28
Adverse event leading to treatment discontinuation	1	19/397	4.79%	7/400	1.75%	2.73 [1.16, 6.43]	0.02
During the Adjuvant Treatment Phase							
Total adverse events	2	182/454	40.09%	88/429	20.51%	1.97 [1.58, 2.46]	<0.00001
Grade 3-5 adverse events	1	29/397	7.30%	15/400	3.75%	1.95 [1.06, 3.58]	0.03
Serious adverse events	1	16/397	4.03%	7/400	1.75%	2.30 [0.96, 5.54]	0.06
Fatal adverse events	1	1/397	0.25%	0/400	0.00%	3.02 [0.12, 73.97]	0.50

CI, confidence interval; P, Probability; PIO, Perioperative immunotherapy; PP, Perioperative placebo.

In the neoadjuvant treatment phase, total AEs, grade 3-5 AEs, serious AEs, and fatal AEs tended to favor the PP group without a significant difference (**Table 2**, **Supplementary Figure S7**). More cases of rash, pruritus, increased alanine aminotransferase, hypothyroidism, and pneumonitis were found in the PIO group (**Supplementary Table S5**). There was no significant difference in the incidence of all grade 3-5 adverse events between the two groups in the neoadjuvant treatment phase (**Supplementary Table S6**).

In the surgical treatment phase, total AEs, grade 3-5 AEs, serious AEs, and fatal AEs tended to favor the PP group without a significant difference. PIO treatment was associated with more ALTD (4.79% vs. 1.75%, RR: 2.73 [1.16, 6.43]) (**Table 2**, **Supplementary Figure S8**). More diarrhea of any grade was found in the PIO group (**Supplementary Table S7**). There was no significant difference in the incidence of all grade 3-5 adverse events between the two groups in the surgical treatment phase (**Supplementary Table S8**).

In the adjuvant treatment phase, PIO treatment resulted in a greater incidence of total AEs (40.09% vs. 20.51%, RR: 1.97 [1.58, 2.46]) and grade 3-5 AEs (7.30% vs. 3.75%, RR: 1.95 [1.06, 3.58]). Serious AEs and fatal AEs tended to favor the PP group, but the difference was not significant (**Table 2**, **Supplementary Figure S9**). More grade pruritus, rash, and hypothyroidism were found in the PIO group (**Supplementary Table S9**). There was no significant difference in the incidence of all grade 3-5 adverse events between the two groups in the adjuvant treatment phase (**Supplementary Table S10**).

### Sensitivity analysis

Analysis of ORR, surgery rate, and R0 resection rate revealed significant heterogeneity. Excluding any study did not affect the stability or reliability of the results, as indicated by the sensitivity analysis (**Supplementary Figure S10**).

## Publication bias

**Figure 6:**
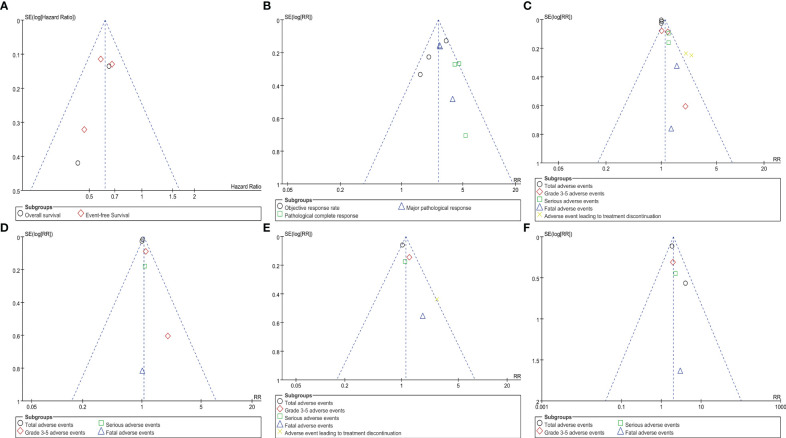
Funnel plots of survival summary **(A)**, pathological responses **(B)**, adverse events’ summary during all treatment phase **(C)**, adverse events’ summary during the neoadjuvant treatment phase **(D)**, adverse events’ summary during the surgical treatment phase **(E)**, adverse events’ summary during the adjuvant treatment phase **(F)** associated with perioperative immunotherapy versus perioperative placebo according to survival time.

Symmetrical funnel plots were observed for survival summary (**Figure 6A**), pathological responses (**Figure 6B**), and AEs (**Figures 6C-F**), indicating acceptable publication bias.

## Discussion

Resectable stage II-III NSCLC cases can have improved outcomes if neoadjuvant and/or adjuvant treatment is given in addition to surgery (20–22). However, although traditional PBC can improve patient survival, it is very limited (23, 24). In recent years, the introduction of immunotherapy in neoadjuvant therapy and adjuvant therapy for resectable NSCLC has brought new hope to the long-term survival of these patients (9–15). This study represents the first meta-analysis analyzing the perioperative use (neoadjuvant+adjuvant) of immunotherapy for stage II-III NSCLC based on RCTs. The results suggested that PIO exhibited superior efficacy in OS, EFS, ORR, PCR, MPR, R0 resection rate, and rate of adjuvant treatment compared with PP. In safety assessment, more serious AEs and ALTD were found in the PIO group.

The primary advantage of PIO treatment lies in improved survival, particularly in terms of OS. In this study, the HR for survival was 0.63 [0.49-0.81] for OS and 0.61 [0.52, 0.72] for EFS. EFS is currently the primary endpoint in most RCTs on the perioperative treatment of NSCLC. In neoadjuvant therapy, the HR of EFS was 0.63 [0.43-0.91] in the CheckMate 816 study (9). In adjuvant therapy, the HR of EFS was 0.66 [0.50-0.88] in the Impower 010 study and 0.76 [0.63-0.91] in the KEYNOTE-091 study (11, 12). In addition, the Neotorch study (toripalimab) has reported interim research results with EFS (HR, 0.40 [0. 277-0. 565]) in ASCO 2023 (25). Thus, many scholars believed that the combined use of immunotherapy during the perioperative period might bring more survival benefits to patients than using neoadjuvant therapy and adjuvant therapy alone (8, 26). Meanwhile, this study also confirmed that PIO demonstrated an increasing advantage in survival (OS, EFS) compared to PP, which was consistent with the tail effect of immunotherapy (27). In the subgroup analysis, EFS tended to favor the PIO group in almost all subgroups. BMI (>25), T stage (IV), N stage (N1-N2) and pathological response (with PCR) were favorable factors in the PIO group, as substantiated in several studies (28, 29). Additionally, the EFS advantage of the PIO group increased with increasing PD-L1 expression (PD-L1 TPS, < 1%, RR: 0.77 [0.59-1.00]; 1-49%, RR: 0.56 [0.42-0.73]; > 50%, RR: 0.48 [0.35-0.67]).

Neoadjuvant immunotherapy may have improved survival benefits, although a direct comparative randomized trial would need to be conducted to determine this (30, 31). Therefore, the pathological response and its impact on surgical treatment are crucial indicators for evaluating drug efficacy. In summary, the ORR, PCR and MPR were 51.46%, 19.02% and 32.44% in the PIO group, which was similar to the results of NADIM study and SAKK 16/14 study (32, 33). In this study, patients in the PIO group achieved better ORR (RR: 2.21 [1.91, 2.54]), PCR (RR: 4.36 [3.04, 6.25]) and MPR (RR: 2.79 [2.25, 3.46]) compared to patients in the PP group. Similar results were also confirmed by the CheckMate 816 study and the Neotorch study (9, 25). Better pathological response was also associated with increased surgery rate (82.07% vs. 79.58%) and R0 resection rate (75.24% vs. 67.87%), playing a crucial role in the long-term survival of patients. Furthermore, we confirmed that the EFS advantage in the PIO group was particularly notable in the PCR subgroup. Therefore, it can be indirectly confirmed that a better pathological response could lead to a better prognosis in perioperative immunotherapy.

Safety is another concern in the perioperative and long-term use of immunotherapy after surgery. The IMpower010 trial reported that Atezolizumab-related adverse events leading to hospitalization occurred in 7% of the surgery groups (34). In clinical practice, although the incidence of AEs in immunotherapy is often much lower than that in chemotherapy, immune related AEs (such as pneumonitis, myocarditis, etc.) are often challenging to manage and can substantially impact the quality of life (35). At different periods of this study, it was observed that the incidence of total AEs, grade 3-5 AEs, serious AEs, and fatal AEs was higher in the PIO group than in the PP group in varying degrees, especially during the neoadjuvant treatment phase. In this phase, the top 5 AEs in the PIO group were nausea (41.15%), anemia (36.17%), neutrophil count decreased (30.28%), constipation (26.87%), and fatigue (23.17%), similar to those in the PP group. These common AEs are often associated with chemotherapy (13). The incidences of rash, pruritus, alanine aminotransferase increased, hypothyroidism, and pneumonitis were significant higher in the PIO group. These significantly increased AEs are often associated with immunotherapy (36). Therefore, although PIO can substantially improve survival, the monitoring and treatment of AEs at different phases still requires close attention.

This meta-analysis has limitations. Firstly, the inclusion of only English articles may introduce language bias. Secondly, including only 3 RCTs may reduce the overall clinical value. Thirdly, all the data analyzed were extracted from previously published articles, leading to increased data heterogeneity. Fourthly, the absence of individual patient data prevented a meta-analysis at the patient level, potentially decreasing the clinical value. Fifthly, variations in median follow-up times across studies might contribute to increased data heterogeneity.

## Conclusion

PIO appears superior to PP for resectable stage II-III NSCLC, exhibiting better survival (OS and EFS) and improved pathological responses. Survival tended to favor the PIO group across almost all subgroups. Additionally, PIO demonstrated an increased advantage in survival compared to PP with longer follow up and increased PD-L1 expression. However, the higher rate of AEs in the PIO group warrants serious consideration.

## Data availability statement

The original contributions presented in the study are included in the article/**Supplementary Material**. Further inquiries can be directed to the corresponding author.

## Author contributions

AY: Conceptualization, Data curation, Formal analysis, Investigation, Methodology, Project administration, Resources, Software, Supervision, Validation, Visualization, Writing – original draft, Writing – review & editing. FF: Conceptualization, Data curation, Formal analysis, Writing – original draft, Writing – review & editing. XL: Conceptualization, Data curation, Formal analysis, Writing – original draft, Writing – review & editing. MW: Conceptualization, Data curation, Formal analysis, Writing – original draft, Writing – review & editing. MY: Conceptualization, Data curation, Formal analysis, Funding acquisition, Investigation, Methodology, Project administration, Resources, Software, Supervision, Validation, Visualization, Writing – original draft, Writing – review & editing. WZ: Conceptualization, Data curation, Formal analysis, Funding acquisition, Writing – original draft, Writing – review & editing.

## Supplementary material

Supplementary Figure 1 Cochrane Risk Assessment.

Supplementary Figure 2 Comparisons of overall survival rate (6-48 months) associated with perioperative immunotherapy versus perioperative placebo according to survival time.

Supplementary Figure 3 Comparisons of event-free survival rate (6-48 months) associated with perioperative immunotherapy versus perioperative placebo according to survival time.

Supplementary Figure 4 Forest plots of surgery rate and R0 resection rate associated with perioperative immunotherapy versus perioperative placebo according to survival time.

Supplementary Figure 5 Treatment summary of adjuvant phase.

Supplementary Figure 6 Forest plots of adverse events’ summary during all treatment phase associated with perioperative immunotherapy versus perioperative placebo.

Supplementary Figure 7 Forest plots of adverse events’ summary during the neoadjuvant treatment phase associated with perioperative immunotherapy versus perioperative placebo.

Supplementary Figure 8 Forest plots of adverse events’ summary during the surgical treatment phase associated with perioperative immunotherapy versus perioperative placebo.

Supplementary Figure 9 Forest plots of adverse events’ summary during the adjuvant treatment phase associated with perioperative immunotherapy versus perioperative placebo.

Supplementary Figure 10 Sensitivity analysis of objective response rate **(A)**, surgery rate **(B)**, and R0 resection rate **(C)**.
