# Supplementary figures and images for: Perioperative immunotherapy for stage II-III non-small cell lung cancer: a meta-analysis base on randomized controlled trials
Source: Front Oncol. 2024 Feb 22;14:1351359. doi: 10.3389/fonc.2024.1351359 (PMC10917905; doi:10.3389/fonc.2024.1351359)

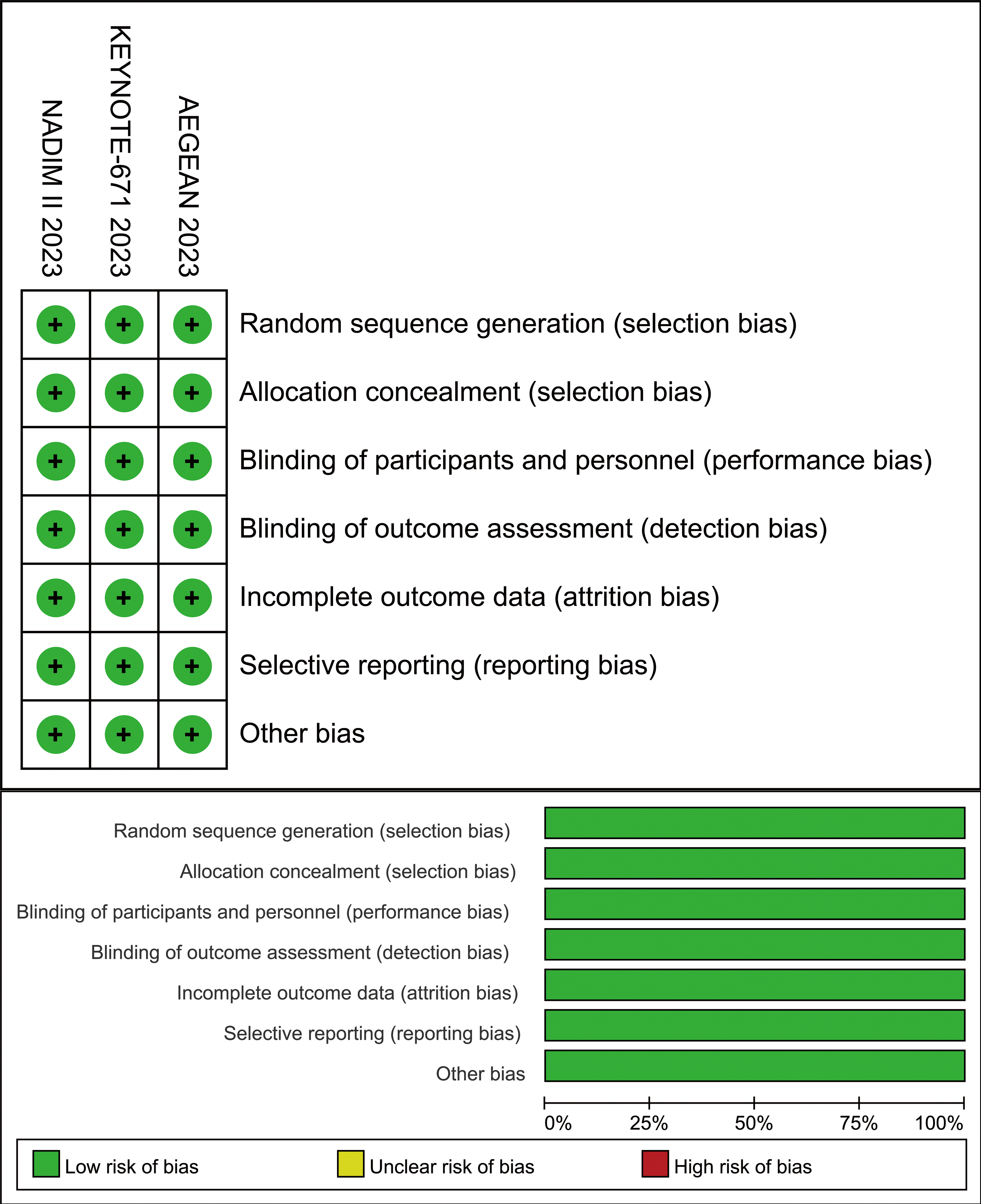

Supplement: Supplementary Figure 1 — Cochrane Risk Assessment. [file Image_1.tif]

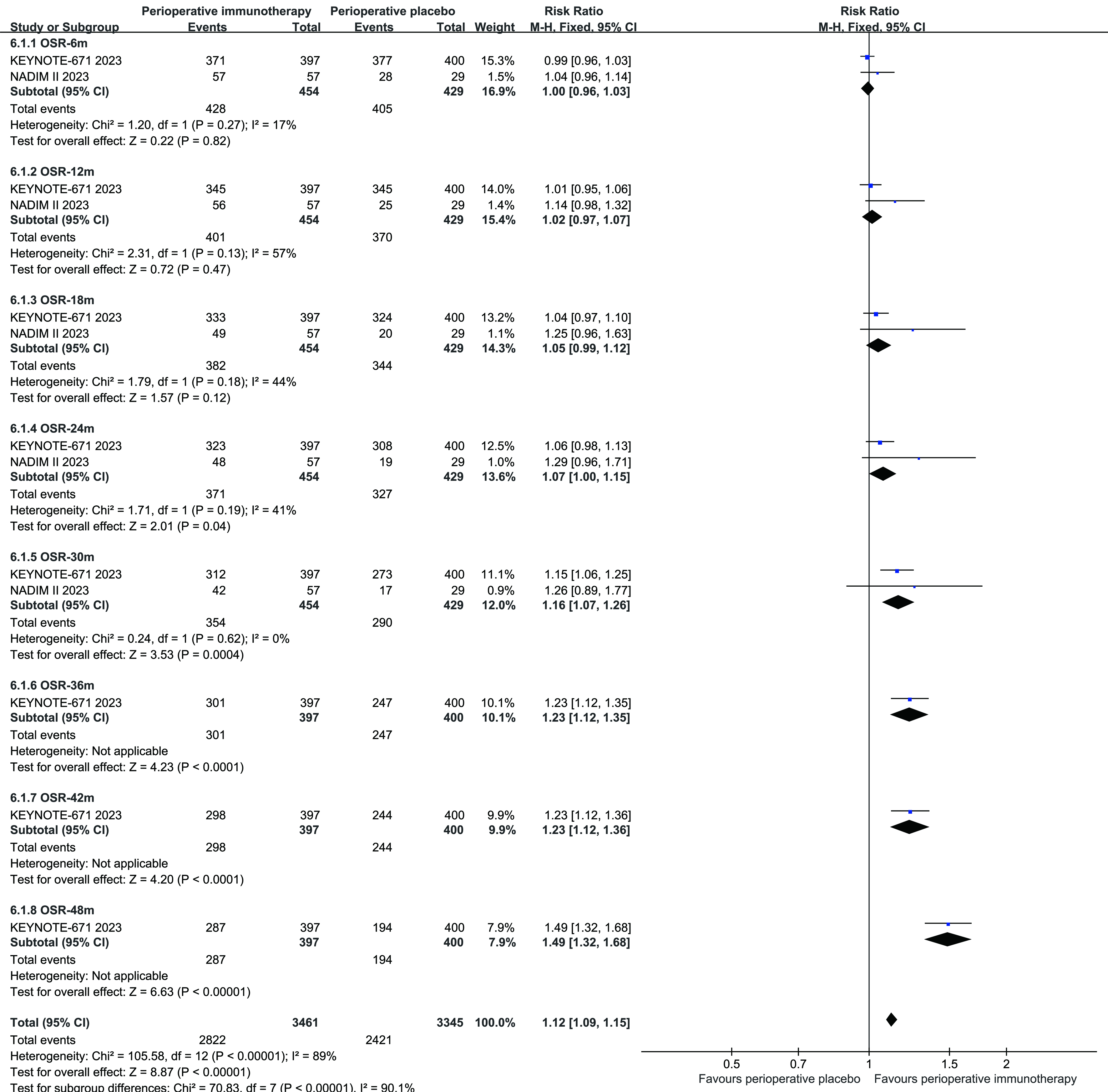

Supplement: Supplementary Figure 2 — Comparisons of overall survival rate (6-48 months) associated with perioperative immunotherapy versus perioperative placebo according to survival time. [file Image_2.tif]

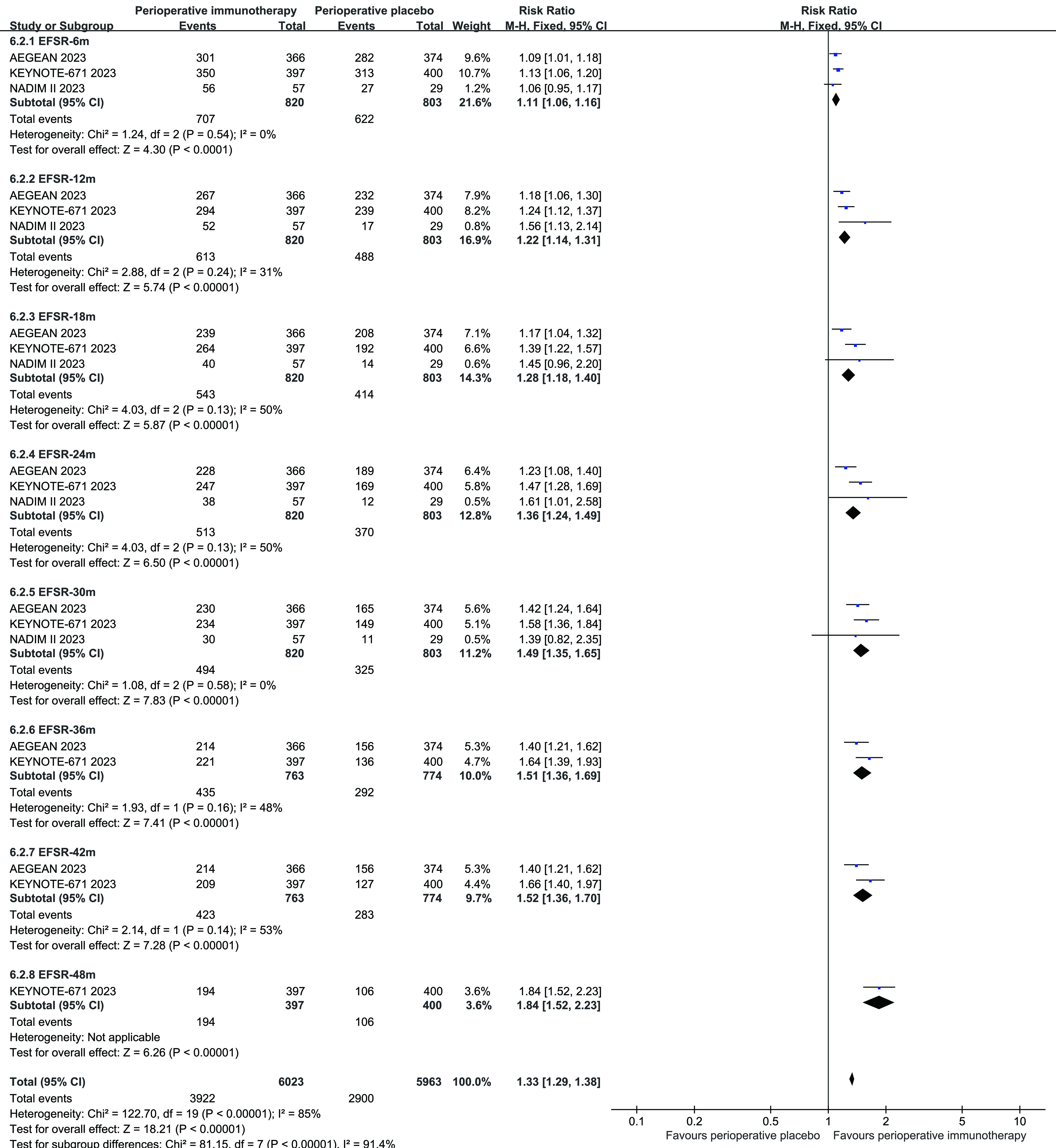

Supplement: Supplementary Figure 3 — Comparisons of event-free survival rate (6-48 months) associated with perioperative immunotherapy versus perioperative placebo according to survival time. [file Image_3.tif]

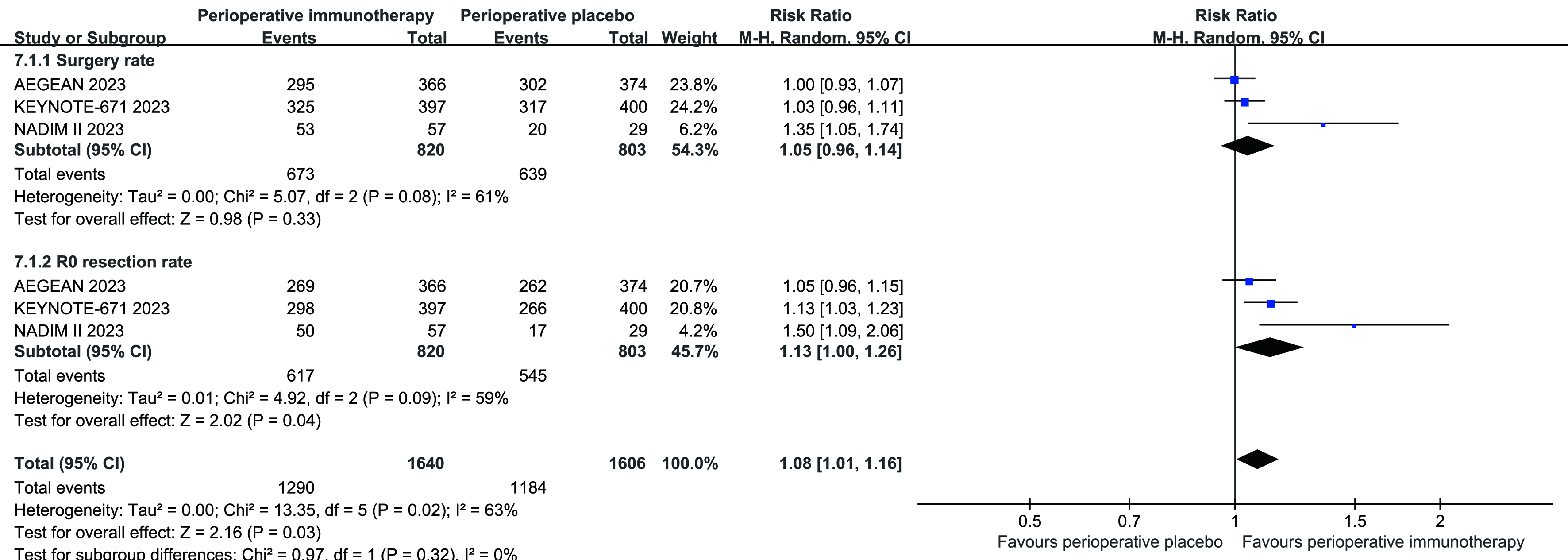

Supplement: Supplementary Figure 4 — Forest plots of surgery rate and R0 resection rate associated with perioperative immunotherapy versus perioperative placebo according to survival time. [file Image_4.tif]

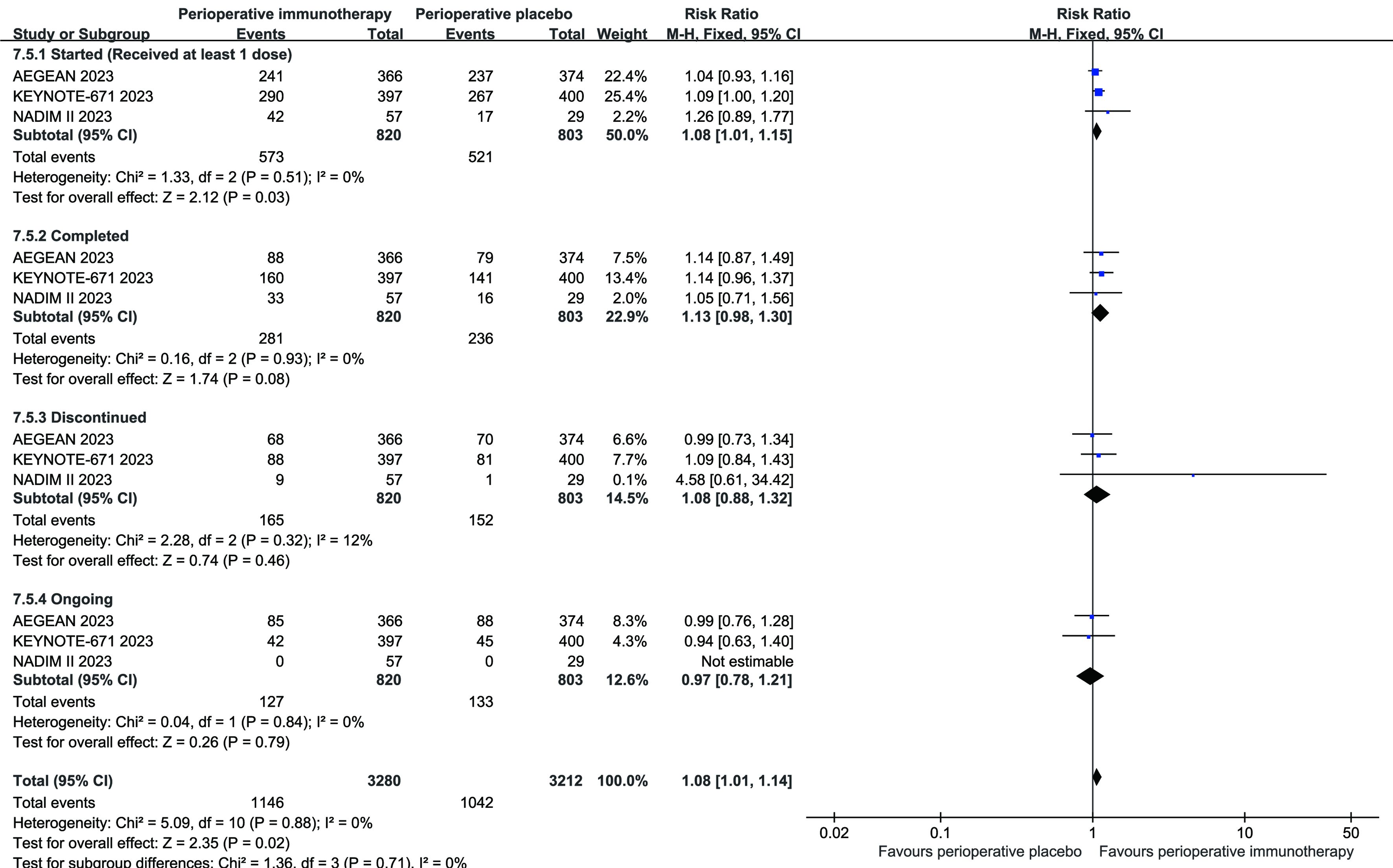

Supplement: Supplementary Figure 5 — Treatment summary of adjuvant phase. [file Image_5.tif]

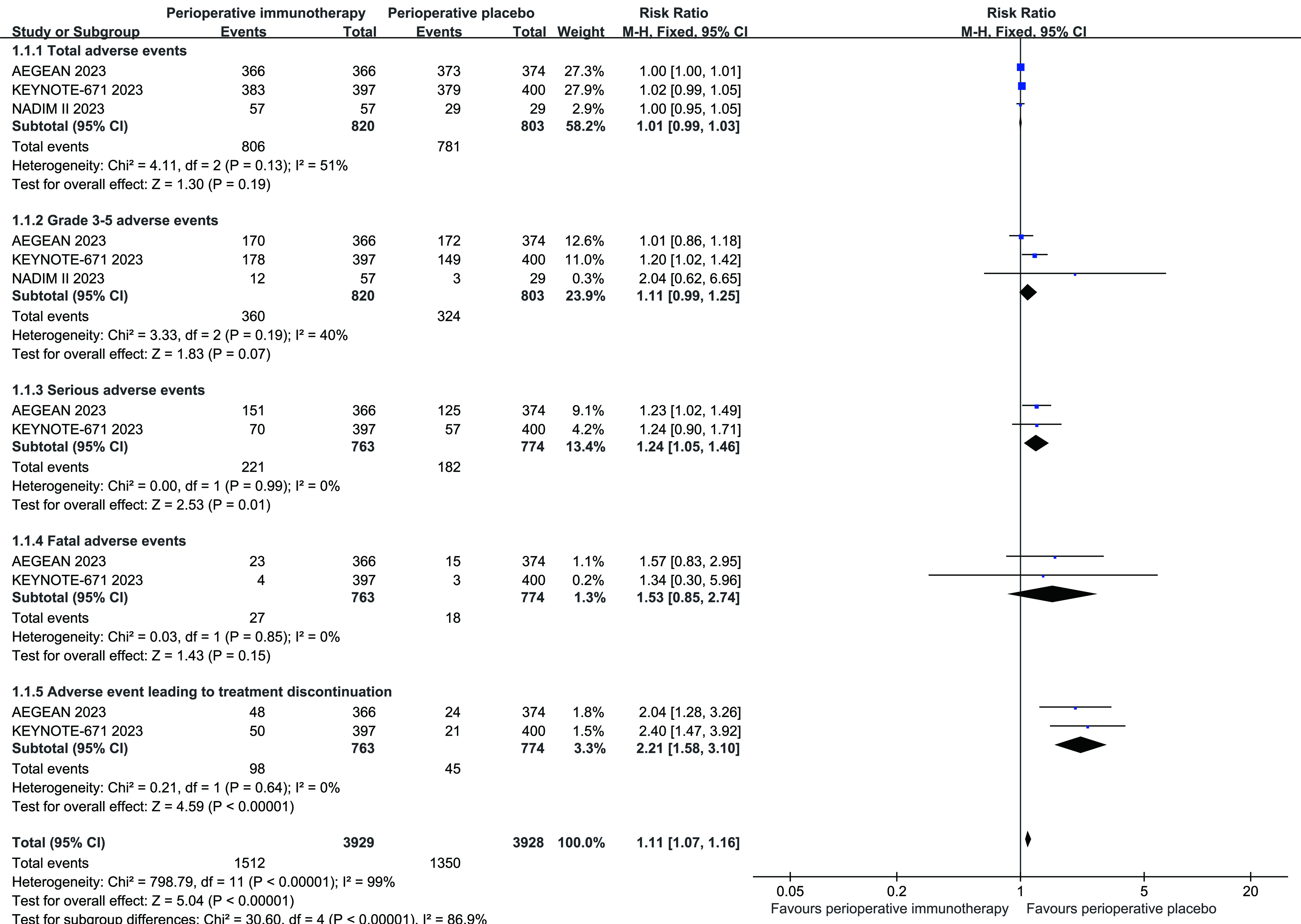

Supplement: Supplementary Figure 6 — Forest plots of adverse events’ summary during all treatment phase associated with perioperative immunotherapy versus perioperative placebo. [file Image_6.tif]

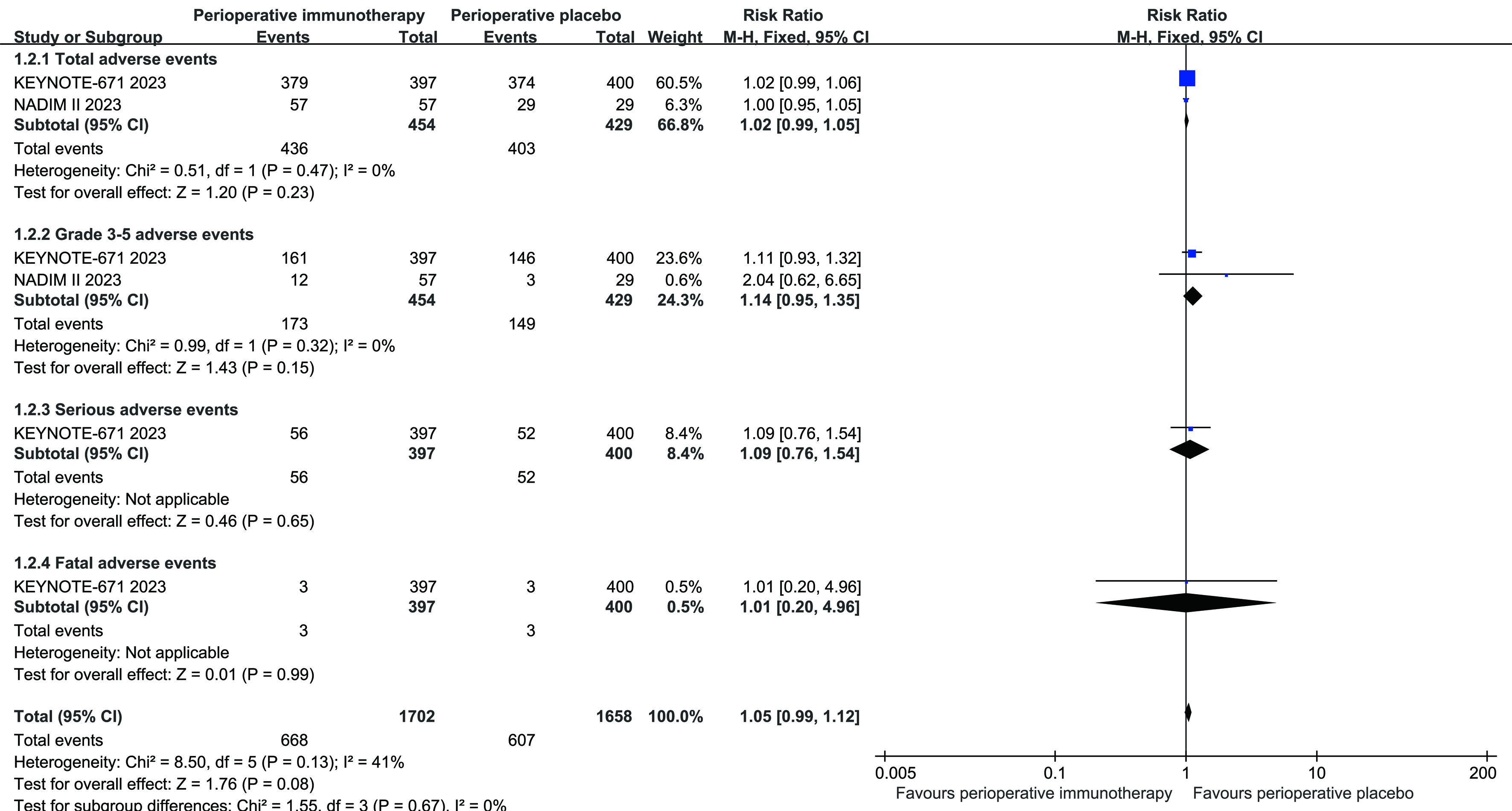

Supplement: Supplementary Figure 7 — Forest plots of adverse events’ summary during the neoadjuvant treatment phase associated with perioperative immunotherapy versus perioperative placebo. [file Image_7.tif]

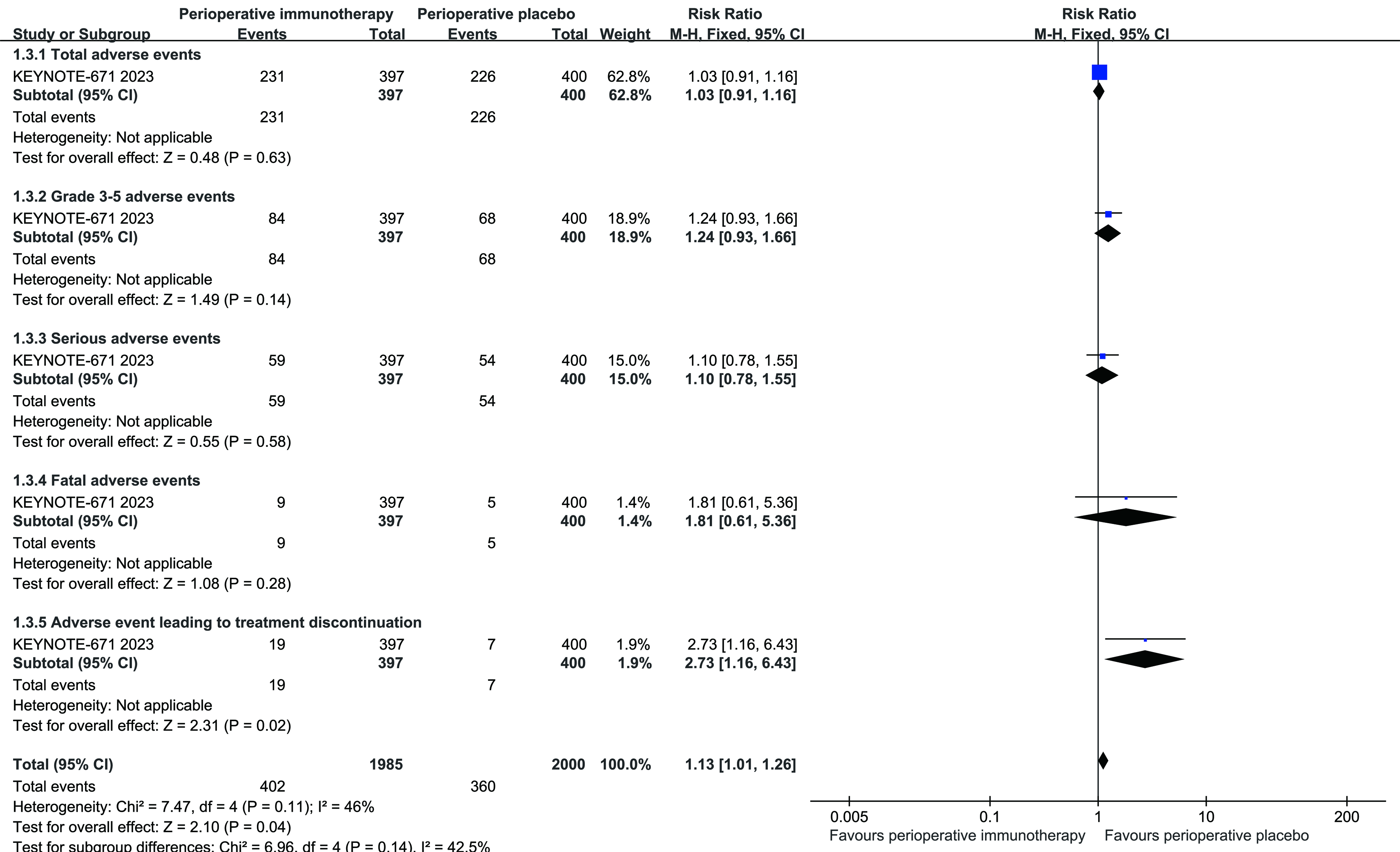

Supplement: Supplementary Figure 8 — Forest plots of adverse events’ summary during the surgical treatment phase associated with perioperative immunotherapy versus perioperative placebo. [file Image_8.tif]

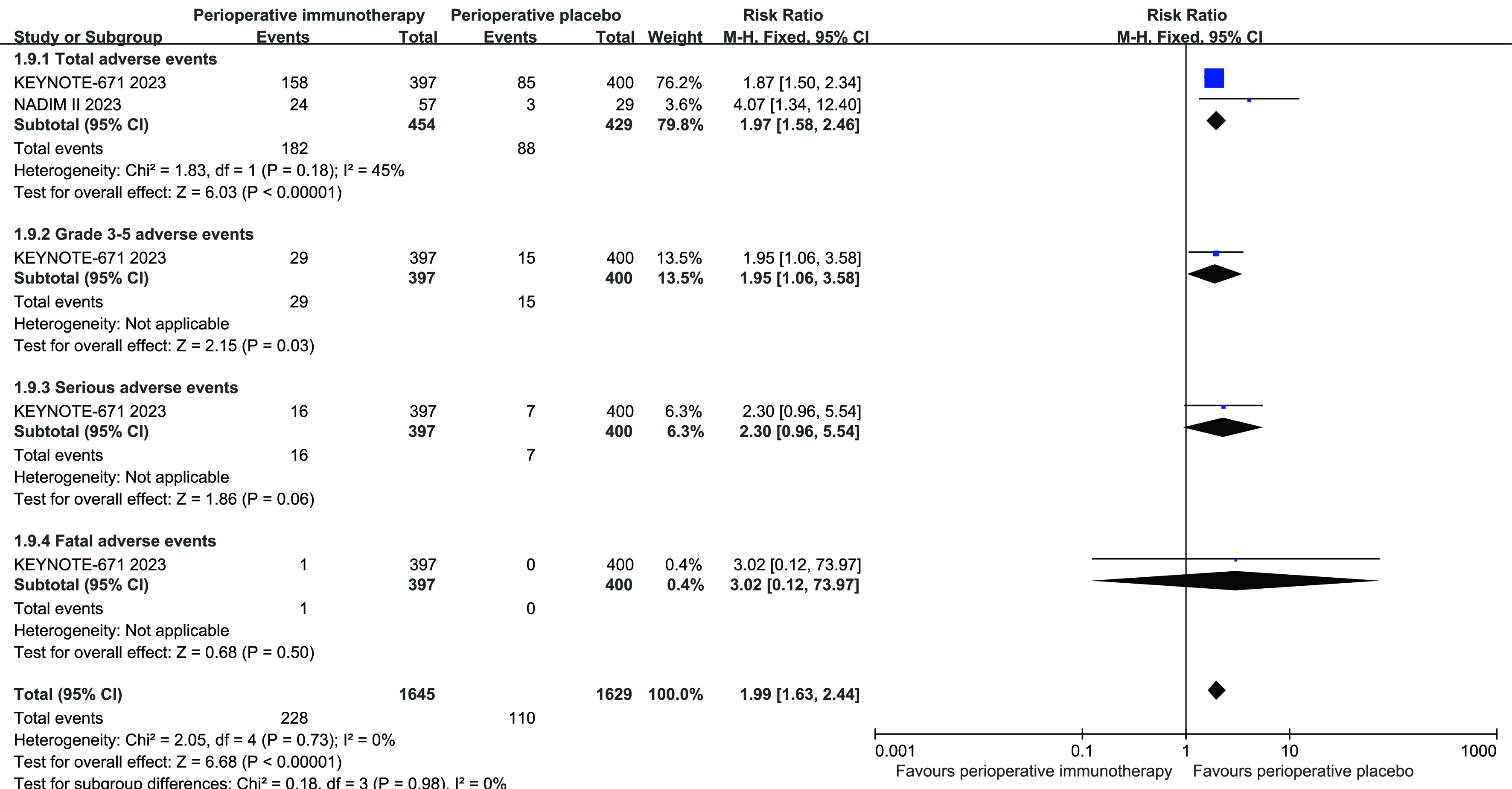

Supplement: Supplementary Figure 9 — Forest plots of adverse events’ summary during the adjuvant treatment phase associated with perioperative immunotherapy versus perioperative placebo. [file Image_9.tif]

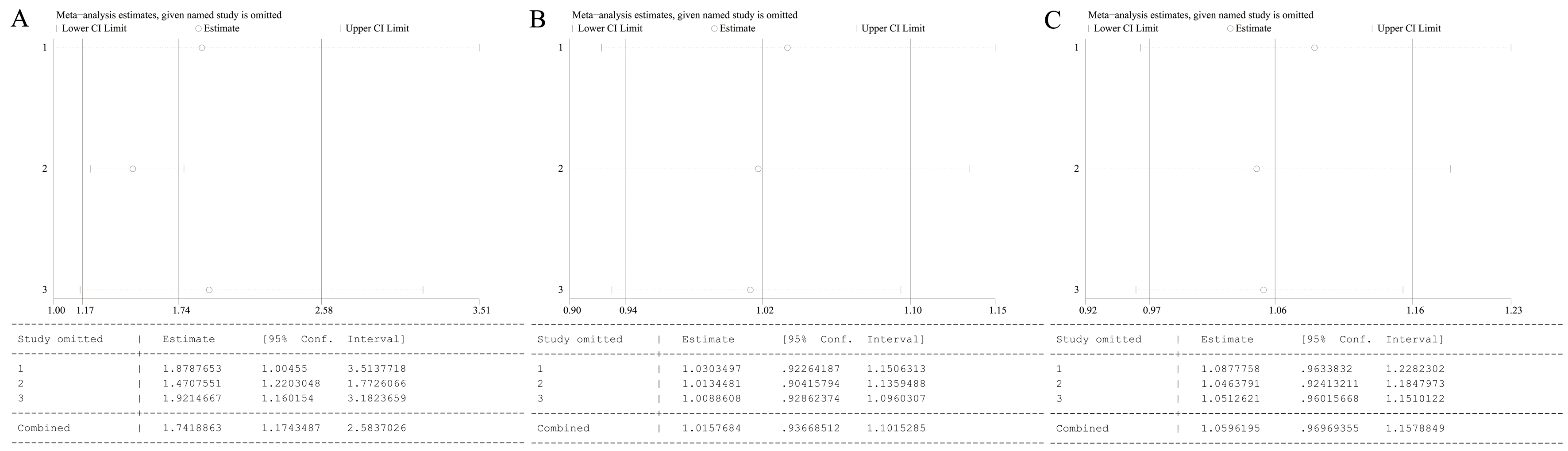

Supplement: Supplementary Figure 10 — Sensitivity analysis of objective response rate (A), surgery rate (B), and R0 resection rate (C). [file Image_10.tif]
