# Supplementary material for: Perioperative immunotherapy for stage II-III non-small cell lung cancer: a meta-analysis base on randomized controlled trials
Source: Front Oncol. 2024 Feb 22;14:1351359. doi: 10.3389/fonc.2024.1351359 (PMC10917905; doi:10.3389/fonc.2024.1351359)
Supplement: Supplementary file 12 [file Table_2.docx]

**Table S2** Search strategy

| **PubMed**  The database was searched on November 15, 2023, n=593.  Search Strategy:  **(((((((((((((Nivolumab[Title/Abstract]) OR (Pembrolizumab[Title/Abstract])) OR (Treprinumab[Title/Abstract])) OR (Cedilimumab[Title/Abstract])) OR (Camrelizumab[Title/Abstract])) OR (Tislelizumab[Title/Abstract])) OR (Penpulimab[Title/Abstract])) OR (Zimberelimab[Title/Abstract])) OR (serplulimab[Title/Abstract])) OR (Durvalumab[Title/Abstract])) OR (Atezolizumab[Title/Abstract])) OR (Envolizumab[Title/Abstract])) OR (Sugemalimab[Title/Abstract])) OR (Tremelimumab[Title/Abstract])) OR (Lpilimumab [Title/Abstract])) OR (Adebrelimab[Title/Abstract]) AND (Lung cancer [Title/Abstract])** |
| --- |
| **Web of Science**  The database was searched on November 15, 2023, n=580.  Search Strategy:  (Nivolumab or Pembrolizumab or Treprinumab or Cedilimumab or Camrelizumab or Tislelizumab or Penpulimab or Zimberelimab or serplulimab or Durvalumab or Atezolizumab or Envolizumab or Sugemalimab or Adebrelimab or Tremelimumab or Lpilimumab (Abstract)) AND Lung cancer (Abstract) AND (Randomized or Randomly or Randomised (Abstract)) |
| **EMBASE**  The database was searched on November 15, 2023, n=1567.  Search Strategy:  (Nivolumab or Pembrolizumab or Treprinumab or Cedilimumab or Camrelizumab or Tislelizumab or Penpulimab or Zimberelimab or serplulimab or Durvalumab or Atezolizumab or Envolizumab or Sugemalimab or Adebrelimab or Tremelimumab or Lpilimumab):ti,ab,kw AND (Lung cancer):ti,ab,kw AND (Randomized or Randomly or Randomised):ti,ab,kw" |
| **Cochrane Library**  The database was searched on November 15, 2023, n=1969.  Search Strategy:  (Nivolumab or Pembrolizumab or Treprinumab or Cedilimumab or Camrelizumab or Tislelizumab or Penpulimab or Zimberelimab or serplulimab or Durvalumab or Atezolizumab or Envolizumab or Sugemalimab or Adebrelimab or Tremelimumab or Lpilimumab):ti,ab,kw AND (Lung cancer):ti,ab,kw AND (Randomized or Randomly or Randomised):ti,ab,kw" (Word variations have been searched) |
| **Ovid MEDLINE**  The database was searched on November 15, 2023, n=239.  Search Strategy:  **(((((((((((((Nivolumab) OR (Pembrolizumab)) OR (Treprinumab)) OR (Cedilimumab)) OR (Camrelizumab)) OR (Tislelizumab)) OR (Penpulimab)) OR (Zimberelimab)) OR (serplulimab)) OR (Durvalumab)) OR (Atezolizumab)) OR (Envolizumab)) OR (Sugemalimab)) OR (**Tremelimumab**)) OR (**Lpilimumab**)) OR (Adebrelimab) and (Lung cancer) and (((Randomized) OR (Randomised)) OR Randomly)** |
| **ScienceDirect**  The database was searched on November 15, 2023, n=1325.  Search Strategy:  Title, abstract, keywords: ((“nivolumab” OR “pembrolizumab” OR “treprinumab” OR “cedilimumab” OR “camrelizumab” OR “tislelizumab” OR “penpulimab” OR “zimberelimab” OR “serplulimab” OR “durvalumab” OR “atezolizumab” OR “envolizumab” OR “sugemalimab” OR “tremelimumab” OR “lpilimumab” OR “adebrelimab”) and (“lung cancer”) and (“randomized” OR “randomly” OR “randomised”)) |
| **Scopus**  The database was searched on November 15, 2023, n=2,268.  Search Strategy:  (TITLE-ABS-KEY (nivolumab OR pembrolizumab OR treprinumab OR cedilimumab OR camrelizumab OR tislelizumab OR penpulimab OR zimberelimab OR serplulimab OR durvalumab OR atezolizumab OR envolizumab OR sugemalimab OR tremelimumab OR lpilimumab OR adebrelimab) AND TITLE-ABS-KEY (lung AND cancer) AND TITLE-ABS-KEY (randomized OR randomly OR randomised)) |

**Note:** The combined text and medical subject heading (MeSH) terms used were: “lung cancer”, “randomized” and immune checkpoint inhibitors (including Nivolumab, Pembrolizumab, Treprinumab, Cedilimumab, Camrelizumab, Tislelizumab, Penpulimab, Zimberelimab, serplulimab, Durvalumab, Atezolizumab, Envolizumab, Sugemalimab, Adebrelimab, Ipilimumab and Tremelimumab).
