# Supplementary material for: Perioperative immunotherapy for stage II-III non-small cell lung cancer: a meta-analysis base on randomized controlled trials
Source: Front Oncol. 2024 Feb 22;14:1351359. doi: 10.3389/fonc.2024.1351359 (PMC10917905; doi:10.3389/fonc.2024.1351359)
Supplement: Supplementary file 13 [file Table_3.docx]

**Table S3** Quality assessment of the included studies according to the Jadad scale.

| **Study** | | | **Randomization** | **Masking** | **Accountability of all patients** | **Quality (score)** |
| --- | --- | --- | --- | --- | --- | --- |
| KEYNOTE-671 | NCT03425643 | Wakelee 2023 [13] | ** | ** | * | 5 |
| NADIM II | NCT03838159 | Provencio 2023 [14] | ** | ** | * | 5 |
| AEGEAN | NCT03800134 | Heymach 2023 [15] | ** | ** | * | 5 |
