# Supplementary material for: Perioperative immunotherapy for stage II-III non-small cell lung cancer: a meta-analysis base on randomized controlled trials
Source: Front Oncol. 2024 Feb 22;14:1351359. doi: 10.3389/fonc.2024.1351359 (PMC10917905; doi:10.3389/fonc.2024.1351359)
Supplement: Supplementary file 14 [file Table_4.doc]

**Table S4 GRADE quality assessment for the outcomes of survival, pathological responses, and adverse events.**

| **Primary outcomes** | **No. of Studies** | **No. of Participants** | | **Differences (95%CI) ^a^** | **Quality Assessment** | | | | | **Quality** |
| --- | --- | --- | --- | --- | --- | --- | --- | --- | --- | --- |
|  |  | **PIO** | **PP** |  | **Risk of Bias^b^** | **Inconsistency** | **Indirectness** | **Imprecision** | **Publication Bias^c^** |  |
| **Survival** |  |  |  |  |  |  |  |  |  |  |
| OS | 2 | 454 | 429 | 0.63 [0.49, 0.81] | Low | No inconsistency | No indirectness | No imprecision | Unlikely | High |
| OSR |  |  |  |  |  |  |  |  |  |  |
| 6-month | 2 | 428/454 | 405/429 | 1.00 [0.96, 1.03] | Low | No inconsistency | No indirectness | No imprecision | Unlikely | High |
| 12-month | 2 | 401/454 | 370/429 | 1.02 [0.97, 1.07] | Low | No inconsistency | No indirectness | No imprecision | Unlikely | High |
| 18-month | 2 | 382/454 | 344/429 | 1.05 [0.99, 1.12] | Low | No inconsistency | No indirectness | No imprecision | Unlikely | High |
| 24-month | 2 | 371/454 | 327/429 | 1.07 [1.00, 1.15] | Low | No inconsistency | No indirectness | No imprecision | Unlikely | High |
| 30-month | 2 | 354/454 | 290/429 | 1.16 [1.07, 1.26] | Low | No inconsistency | No indirectness | No imprecision | Unlikely | High |
| 36-month | 1 | 301/397 | 247/400 | 1.23 [1.12, 1.35] | Low | No inconsistency | No indirectness | No imprecision | Unlikely | High |
| 42-month | 1 | 298/397 | 244/400 | 1.23 [1.12, 1.36] | Low | No inconsistency | No indirectness | No imprecision | Unlikely | High |
| 48-month | 1 | 287/397 | 194/400 | 1.49 [1.32, 1.68] | Low | No inconsistency | No indirectness | No imprecision | Unlikely | High |
| EFS | 3 | 820 | 803 | 0.61 [0.52, 0.72] | Low | No inconsistency | No indirectness | No imprecision | Unlikely | High |
| EFSR |  |  |  |  |  |  |  |  |  |  |
| 6-month | 3 | 707/820 | 622/803 | 1.11 [1.06, 1.16] | Low | No inconsistency | No indirectness | No imprecision | Unlikely | High |
| 12-month | 3 | 613/820 | 488/803 | 1.22 [1.14, 1.31] | Low | No inconsistency | No indirectness | No imprecision | Unlikely | High |
| 18-month | 3 | 543/820 | 414/803 | 1.28 [1.18, 1.40] | Low | No inconsistency | No indirectness | No imprecision | Unlikely | High |
| 24-month | 3 | 513/820 | 370/803 | 1.36 [1.24, 1.49] | Low | No inconsistency | No indirectness | No imprecision | Unlikely | High |
| 30-month | 3 | 494/820 | 325/803 | 1.49 [1.35, 1.65] | Low | No inconsistency | No indirectness | No imprecision | Unlikely | High |
| 36-month | 2 | 435/763 | 292/774 | 1.51 [1.36, 1.69] | Low | No inconsistency | No indirectness | No imprecision | Unlikely | High |
| 42-month | 2 | 423/763 | 283/774 | 1.52 [1.36, 1.70] | Low | No inconsistency | No indirectness | No imprecision | Unlikely | High |
| 48-month | 1 | 194/397 | 106/400 | 1.84 [1.52, 2.23] | Low | No inconsistency | No indirectness | No imprecision | Unlikely | High |
| **Pathological responses** |  |  |  |  |  |  |  |  |  |  |
| Objective response rate | 3 | 422/820 | 183/803 | 2.21 [1.91, 2.54] | Low | No inconsistency | No indirectness | No imprecision | Unlikely | High |
| Pathological complete response | 3 | 156/820 | 34/803 | 4.36 [3.04, 6.25] | Low | No inconsistency | No indirectness | No imprecision | Unlikely | High |
| Major pathological response | 3 | 272/820 | 94/803 | 2.79 [2.25, 3.46] | Low | No inconsistency | No indirectness | No imprecision | Unlikely | High |
| **Surgery summary** |  |  |  |  |  |  |  |  |  |  |
| Surgery rate | 3 | 673/820 | 639/803 | 1.05 [0.96, 1.14] | Low | Serious (-1) | No indirectness | No imprecision | Unlikely | Medium |
| R0 resection rate | 3 | 617/820 | 545/803 | 1.13 [1.00, 1.26] | Low | Serious (-1) | No indirectness | No imprecision | Unlikely | Medium |
| **Adverse events summary during all phases** | | |  |  |  |  |  |  |  |  |
| Total adverse events | 3 | 806/820 | 781/803 | 1.01 [0.99, 1.03] | Low | No inconsistency | No indirectness | No imprecision | Unlikely | High |
| Grade 3-5 adverse events | 3 | 360/820 | 324/803 | 1.11 [0.99, 1.25] | Low | No inconsistency | No indirectness | No imprecision | Unlikely | High |
| Serious adverse events | 2 | 221/763 | 182/774 | 1.24 [1.05, 1.46] | Low | No inconsistency | No indirectness | No imprecision | Unlikely | High |
| Fatal adverse events | 2 | 27/763 | 18/774 | 1.53 [0.85, 2.74] | Low | No inconsistency | No indirectness | No imprecision | Unlikely | High |
| Adverse event leading to treatment discontinuation | 2 | 98/763 | 45/774 | 2.21 [1.58, 3.10] | Low | No inconsistency | No indirectness | No imprecision | Unlikely | High |
| **Adverse events summary during the Neoadjuvant Treatment Phase** | | | |  |  |  |  |  |  |  |
| Total adverse events | 2 | 436/454 | 403/429 | 1.02 [0.99, 1.05] | Low | No inconsistency | No indirectness | No imprecision | Unlikely | High |
| Grade 3-5 adverse events | 2 | 173/454 | 149/429 | 1.14 [0.95, 1.35] | Low | No inconsistency | No indirectness | No imprecision | Unlikely | High |
| Serious adverse events | 1 | 56/397 | 52/400 | 1.09 [0.76, 1.54] | Low | No inconsistency | No indirectness | No imprecision | Unlikely | High |
| Fatal adverse events | 1 | 3/397 | 3/400 | 1.01 [0.20, 4.96] | Low | No inconsistency | No indirectness | No imprecision | Unlikely | High |
| **Adverse events summary during the Surgical Treatment Phase** | | | |  |  |  |  |  |  |  |
| Total adverse events | 1 | 231/397 | 226/400 | 1.03 [0.91, 1.16] | Low | No inconsistency | No indirectness | No imprecision | Unlikely | High |
| Grade 3-5 adverse events | 1 | 84/397 | 68/400 | 1.24 [0.93, 1.66] | Low | No inconsistency | No indirectness | No imprecision | Unlikely | High |
| Serious adverse events | 1 | 59/397 | 54/400 | 1.10 [0.78, 1.55] | Low | No inconsistency | No indirectness | No imprecision | Unlikely | High |
| Fatal adverse events | 1 | 9/397 | 5/400 | 1.81 [0.61, 5.36] | Low | No inconsistency | No indirectness | No imprecision | Unlikely | High |
| Adverse event leading to treatment discontinuation | 1 | 19/397 | 7/400 | 2.73 [1.16, 6.43] | Low | No inconsistency | No indirectness | No imprecision | Unlikely | High |
| **Adverse events summary during the Adjuvant Treatment Phase** | | | |  |  |  |  |  |  |  |
| Total adverse events | 2 | 182/454 | 88/429 | 1.97 [1.58, 2.46] | Low | No inconsistency | No indirectness | No imprecision | Unlikely | High |
| Grade 3-5 adverse events | 1 | 29/397 | 15/400 | 1.95 [1.06, 3.58] | Low | No inconsistency | No indirectness | No imprecision | Unlikely | High |
| Serious adverse events | 1 | 16/397 | 7/400 | 2.30 [0.96, 5.54] | Low | No inconsistency | No indirectness | No imprecision | Unlikely | High |
| Fatal adverse events | 1 | 1/397 | 0/400 | 3.02 [0.12, 73.97] | Low | No inconsistency | No indirectness | No imprecision | Unlikely | High |
| **Adverse events during the neoadjuvant treatment phase** | | | |  |  |  |  |  |  |  |
| Nausea | 2 | 314/763 | 317/774 | 1.00 [0.89, 1.13] | Low | No inconsistency | No indirectness | No imprecision | Unlikely | High |
| Anemia | 2 | 276/763 | 260/774 | 1.08 [0.94, 1.23] | Low | No inconsistency | No indirectness | No imprecision | Unlikely | High |
| Neutrophil count decreased | 2 | 231/763 | 223/774 | 1.05 [0.90, 1.22] | Low | No inconsistency | No indirectness | No imprecision | Unlikely | High |
| Constipation | 2 | 205/763 | 183/774 | 1.14 [0.96, 1.35] | Low | No inconsistency | No indirectness | No imprecision | Unlikely | High |
| Fatigue | 3 | 190/820 | 150/803 | 1.18 [0.98, 1.43] | Low | No inconsistency | No indirectness | No imprecision | Unlikely | High |
| Peripheral sensory neuropathy | 1 | 13/57 | 10/29 | 0.66 [0.33, 1.32] | Low | No inconsistency | No indirectness | No imprecision | Unlikely | High |
| Decreased appetite | 2 | 159/763 | 156/774 | 1.03 [0.85, 1.26] | Low | No inconsistency | No indirectness | No imprecision | Unlikely | High |
| Myalgia | 1 | 11/57 | 3/29 | 1.87 [0.56, 6.17] | Low | No inconsistency | No indirectness | No imprecision | Unlikely | High |
| Neutropenia | 1 | 68/366 | 71/374 | 0.98 [0.73, 1.32] | Low | No inconsistency | No indirectness | No imprecision | Unlikely | High |
| White blood cell count decreased | 2 | 136/763 | 130/774 | 1.06 [0.86, 1.31] | Low | No inconsistency | No indirectness | No imprecision | Unlikely | High |
| Vomiting | 2 | 119/763 | 99/774 | 1.22 [0.95, 1.56] | Low | No inconsistency | No indirectness | No imprecision | Unlikely | High |
| Rash | 2 | 63/423 | 34/403 | 1.80 [1.21, 2.68] | Low | No inconsistency | No indirectness | No imprecision | Unlikely | High |
| Alopecia | 3 | 122/820 | 113/803 | 1.02 [0.80, 1.29] | Low | No inconsistency | No indirectness | No imprecision | Unlikely | High |
| Blood creatinine increased | 1 | 53/397 | 44/400 | 1.21 [0.83, 1.77] | Low | No inconsistency | No indirectness | No imprecision | Unlikely | High |
| Platelet count decreased | 2 | 101/763 | 105/774 | 0.97 [0.76, 1.25] | Low | No inconsistency | No indirectness | No imprecision | Unlikely | High |
| Pruritus | 2 | 54/423 | 24/403 | 2.14 [1.35, 3.40] | Low | No inconsistency | No indirectness | No imprecision | Unlikely | High |
| Asthenia | 2 | 93/763 | 109/774 | 0.87 [0.67, 1.12] | Low | No inconsistency | No indirectness | No imprecision | Unlikely | High |
| Diarrhea | 3 | 99/820 | 98/803 | 0.99 [0.76, 1.29] | Low | No inconsistency | No indirectness | No imprecision | Unlikely | High |
| Insomnia | 1 | 41/366 | 46/374 | 0.91 [0.61, 1.35] | Low | No inconsistency | No indirectness | No imprecision | Unlikely | High |
| Alanine aminotransferase increased | 2 | 74/763 | 44/774 | 1.70 [1.19, 2.44] | Low | No inconsistency | No indirectness | No imprecision | Unlikely | High |
| Leukopenia | 1 | 34/366 | 30/374 | 1.16 [0.72, 1.85] | Low | No inconsistency | No indirectness | No imprecision | Unlikely | High |
| Arthralgia | 2 | 38/423 | 28/403 | 1.08 [0.68, 1.70] | Low | No inconsistency | No indirectness | No imprecision | Unlikely | High |
| Hypothyroidism | 2 | 67/763 | 11/774 | 6.18 [3.30, 11.61] | Low | No inconsistency | No indirectness | No imprecision | Unlikely | High |
| Febrile neutropenia | 1 | 5/57 | 0/29 | 5.69 [0.33, 99.48] | Low | No inconsistency | No indirectness | No imprecision | Unlikely | High |
| Thrombocytopenia | 1 | 25/366 | 29/374 | 0.88 [0.53, 1.47] | Low | No inconsistency | No indirectness | No imprecision | Unlikely | High |
| Hyperthyroidism | 1 | 15/397 | 6/400 | 2.52 [0.99, 6.43] | Low | No inconsistency | No indirectness | No imprecision | Unlikely | High |
| Pneumonitis | 1 | 11/397 | 3/400 | 3.69 [1.04, 13.14] | Low | No inconsistency | No indirectness | No imprecision | Unlikely | High |
| Gastrointestinal disorders | 1 | 1/57 | 3/29 | 0.17 [0.02, 1.56] | Low | No inconsistency | No indirectness | No imprecision | Unlikely | High |
| Infusion reactions | 1 | 5/397 | 4/400 | 1.26 [0.34, 4.66] | Low | No inconsistency | No indirectness | No imprecision | Unlikely | High |
| Severe skin reactions | 1 | 5/397 | 0/400 | 11.08 [0.61, 199.76] | Low | No inconsistency | No indirectness | No imprecision | Unlikely | High |
| Colitis | 1 | 3/397 | 0/400 | 7.05 [0.37, 136.10] | Low | No inconsistency | No indirectness | No imprecision | Unlikely | High |
| Thyroiditis | 1 | 3/397 | 1/400 | 3.02 [0.32, 28.93] | Low | No inconsistency | No indirectness | No imprecision | Unlikely | High |
| Hepatitis | 1 | 1/397 | 0/400 | 3.02 [0.12, 73.97] | Low | No inconsistency | No indirectness | No imprecision | Unlikely | High |
| Hypophysitis | 1 | 1/397 | 0/400 | 3.02 [0.12, 73.97] | Low | No inconsistency | No indirectness | No imprecision | Unlikely | High |
| Myasthenic syndrome | 1 | 1/397 | 0/400 | 3.02 [0.12, 73.97] | Low | No inconsistency | No indirectness | No imprecision | Unlikely | High |
| Myocarditis | 1 | 1/397 | 0/400 | 3.02 [0.12, 73.97] | Low | No inconsistency | No indirectness | No imprecision | Unlikely | High |
| Myositis | 1 | 1/397 | 0/400 | 3.02 [0.12, 73.97] | Low | No inconsistency | No indirectness | No imprecision | Unlikely | High |
| Oral mucositis | 1 | 0/57 | 3/29 | 0.07 [0.00, 1.38] | Low | No inconsistency | No indirectness | No imprecision | Unlikely | High |
| Paresthesia | 1 | 0/57 | 3/29 | 0.07 [0.00, 1.38] | Low | No inconsistency | No indirectness | No imprecision | Unlikely | High |
| Pancreatitis | 1 | 0/397 | 1/400 | 0.34 [0.01, 8.22] | Low | No inconsistency | No indirectness | No imprecision | Unlikely | High |
| Uveitis | 1 | 0/397 | 1/400 | 0.34 [0.01, 8.22] | Low | No inconsistency | No indirectness | No imprecision | Unlikely | High |
| Vasculitis | 1 | 0/397 | 2/400 | 0.20 [0.01, 4.18] | Low | No inconsistency | No indirectness | No imprecision | Unlikely | High |
| **Adverse events during the surgical treatment phase** | | | |  |  |  |  |  |  |  |
| Procedural pain | 1 | 58/397 | 58/400 | 1.01 [0.72, 1.41] | Low | No inconsistency | No indirectness | No imprecision | Unlikely | High |
| Anemia | 1 | 48/397 | 51/400 | 0.95 [0.66, 1.37] | Low | No inconsistency | No indirectness | No imprecision | Unlikely | High |
| Incision site pain | 2 | 64/763 | 62/774 | 1.06 [0.72, 1.55] | Low | No inconsistency | No indirectness | No imprecision | Unlikely | High |
| Dyspnea | 1 | 33/397 | 20/400 | 1.66 [0.97, 2.85] | Low | No inconsistency | No indirectness | No imprecision | Unlikely | High |
| Cough | 1 | 32/397 | 27/400 | 1.19 [0.73, 1.96] | Low | No inconsistency | No indirectness | No imprecision | Unlikely | High |
| Pneumothorax | 2 | 28/454 | 27/429 | 1.09 [0.38, 3.18] | Low | No inconsistency | No indirectness | No imprecision | Unlikely | High |
| Constipation | 1 | 21/397 | 28/400 | 0.76 [0.44, 1.31] | Low | No inconsistency | No indirectness | No imprecision | Unlikely | High |
| Air Leakage | 1 | 3/57 | 2/29 | 0.76 [0.13, 4.32] | Low | No inconsistency | No indirectness | No imprecision | Unlikely | High |
| Chest pain | 1 | 19/397 | 12/400 | 1.60 [0.78, 3.24] | Low | No inconsistency | No indirectness | No imprecision | Unlikely | High |
| Wound complication | 1 | 18/397 | 18/400 | 1.01 [0.53, 1.91] | Low | No inconsistency | No indirectness | No imprecision | Unlikely | High |
| Pleural effusion | 2 | 20/454 | 20/429 | 1.09 [0.29, 4.07] | Low | No inconsistency | No indirectness | No imprecision | Unlikely | High |
| Diarrhea | 1 | 17/397 | 4/400 | 4.28 [1.45, 12.61] | Low | No inconsistency | No indirectness | No imprecision | Unlikely | High |
| Pneumonia | 1 | 14/397 | 20/400 | 0.71 [0.36, 1.38] | Low | No inconsistency | No indirectness | No imprecision | Unlikely | High |
| Respiratory Insufficiency | 1 | 2/57 | 0/29 | 2.59 [0.13, 52.16] | Low | No inconsistency | No indirectness | No imprecision | Unlikely | High |
| Productive cough | 1 | 12/397 | 18/400 | 0.67 [0.33, 1.38] | Low | No inconsistency | No indirectness | No imprecision | Unlikely | High |
| Subcutaneous emphysema | 2 | 9/454 | 18/429 | 0.48 [0.22, 1.07] | Low | No inconsistency | No indirectness | No imprecision | Unlikely | High |
| Chylothorax | 1 | 1/57 | 0/29 | 1.55 [0.07, 36.95] | Low | No inconsistency | No indirectness | No imprecision | Unlikely | High |
| Arrhythmia | 1 | 1/57 | 2/29 | 0.25 [0.02, 2.69] | Low | No inconsistency | No indirectness | No imprecision | Unlikely | High |
| Atelectasis | 1 | 1/57 | 1/29 | 0.51 [0.03, 7.84] | Low | No inconsistency | No indirectness | No imprecision | Unlikely | High |
| Pulmonary Thromboembolism | 1 | 1/57 | 0/29 | 1.55 [0.07, 36.95] | Low | No inconsistency | No indirectness | No imprecision | Unlikely | High |
| Atrial fibrillation | 1 | 6/397 | 17/400 | 0.36 [0.14, 0.89] | Low | No inconsistency | No indirectness | No imprecision | Unlikely | High |
| **Adverse events during the adjuvant treatment phase** | | | |  |  |  |  |  |  |  |
| Fatigue | 1 | 9/57 | 0/29 | 9.83 [0.59, 163.15] | Low | No inconsistency | No indirectness | No imprecision | Unlikely | High |
| Pruritus | 2 | 28/454 | 6/429 | 4.09 [1.75, 9.55] | Low | No inconsistency | No indirectness | No imprecision | Unlikely | High |
| Arthralgia | 1 | 3/57 | 0/29 | 3.62 [0.19, 67.82] | Low | No inconsistency | No indirectness | No imprecision | Unlikely | High |
| Rash | 1 | 18/397 | 8/400 | 2.27 [1.00, 5.15] | Low | No inconsistency | No indirectness | No imprecision | Unlikely | High |
| Hypothyroidism | 2 | 20/454 | 2/429 | 7.46 [2.07, 26.89] | Low | No inconsistency | No indirectness | No imprecision | Unlikely | High |
| Diarrhea | 2 | 18/454 | 12/429 | 1.38 [0.67, 2.84] | Low | No inconsistency | No indirectness | No imprecision | Unlikely | High |
| Alanine aminotransferase increased | 1 | 2/57 | 0/29 | 2.59 [0.13, 52.16] | Low | No inconsistency | No indirectness | No imprecision | Unlikely | High |
| Anemia | 1 | 2/57 | 0/29 | 2.59 [0.13, 52.16] | Low | No inconsistency | No indirectness | No imprecision | Unlikely | High |
| Myalgia | 1 | 2/57 | 0/29 | 2.59 [0.13, 52.16] | Low | No inconsistency | No indirectness | No imprecision | Unlikely | High |
| Peripheral sensory neuropathy | 1 | 2/57 | 0/29 | 2.59 [0.13, 52.16] | Low | No inconsistency | No indirectness | No imprecision | Unlikely | High |
| Pneumonitis | 1 | 11/397 | 4/400 | 2.77 [0.89, 8.63] | Low | No inconsistency | No indirectness | No imprecision | Unlikely | High |
| Hyperthyroidism | 1 | 8/397 | 0/400 | 17.13 [0.99, 295.75] | Low | No inconsistency | No indirectness | No imprecision | Unlikely | High |
| Severe skin reactions | 1 | 4/397 | 0/400 | 9.07 [0.49, 167.87] | Low | No inconsistency | No indirectness | No imprecision | Unlikely | High |
| Colitis | 1 | 2/397 | 0/400 | 5.04 [0.24, 104.60] | Low | No inconsistency | No indirectness | No imprecision | Unlikely | High |
| Hepatitis | 1 | 2/397 | 2/400 | 1.01 [0.14, 7.12] | Low | No inconsistency | No indirectness | No imprecision | Unlikely | High |
| Adrenal insufficiency | 1 | 1/397 | 0/400 | 3.02 [0.12, 73.97] | Low | No inconsistency | No indirectness | No imprecision | Unlikely | High |
| Hypophysitis | 1 | 1/397 | 0/400 | 3.02 [0.12, 73.97] | Low | No inconsistency | No indirectness | No imprecision | Unlikely | High |
| Guillain-Barré syndrome | 1 | 0/397 | 1/400 | 0.34 [0.01, 8.22] | Low | No inconsistency | No indirectness | No imprecision | Unlikely | High |
| Infusion reactions | 1 | 0/397 | 2/400 | 0.20 [0.01, 4.18] | Low | No inconsistency | No indirectness | No imprecision | Unlikely | High |
| Pancreatitis | 1 | 0/397 | 1/400 | 0.34 [0.01, 8.22] | Low | No inconsistency | No indirectness | No imprecision | Unlikely | High |
| **Grade 3-5 adverse events during the neoadjuvant treatment phase** | | | |  |  |  |  |  |  |  |
| Neutrophil count decreased | 2 | 121/763 | 121/774 | 1.02 [0.81, 1.28] | Low | No inconsistency | No indirectness | No imprecision | Unlikely | High |
| Neutropenia | 1 | 36/366 | 38/374 | 0.97 [0.63, 1.49] | Low | No inconsistency | No indirectness | No imprecision | Unlikely | High |
| Anemia | 2 | 55/763 | 48/774 | 1.16 [0.80, 1.69] | Low | No inconsistency | No indirectness | No imprecision | Unlikely | High |
| Febrile neutropenia | 1 | 4/57 | 0/29 | 4.66 [0.26, 83.62] | Low | No inconsistency | No indirectness | No imprecision | Unlikely | High |
| White blood cell count decreased | 2 | 29/763 | 34/774 | 0.87 [0.53, 1.41] | Low | No inconsistency | No indirectness | No imprecision | Unlikely | High |
| Platelet count decreased | 2 | 27/763 | 36/774 | 0.76 [0.47, 1.25] | Low | No inconsistency | No indirectness | No imprecision | Unlikely | High |
| Leukopenia | 1 | 9/366 | 12/374 | 0.77 [0.33, 1.80] | Low | No inconsistency | No indirectness | No imprecision | Unlikely | High |
| Thrombocytopenia | 1 | 6/366 | 9/374 | 0.68 [0.24, 1.89] | Low | No inconsistency | No indirectness | No imprecision | Unlikely | High |
| Pneumonitis | 1 | 5/397 | 0/400 | 11.08 [0.61, 199.76] | Low | No inconsistency | No indirectness | No imprecision | Unlikely | High |
| Nausea | 2 | 8/763 | 7/774 | 1.15 [0.42, 3.16] | Low | No inconsistency | No indirectness | No imprecision | Unlikely | High |
| Diarrhea | 3 | 8/820 | 6/803 | 1.14 [0.40, 3.27] | Low | No inconsistency | No indirectness | No imprecision | Unlikely | High |
| Vomiting | 2 | 7/763 | 5/774 | 1.44 [0.29, 7.08] | Low | No inconsistency | No indirectness | No imprecision | Unlikely | High |
| Alanine aminotransferase increased | 2 | 7/763 | 3/774 | 2.36 [0.61, 9.09] | Low | No inconsistency | No indirectness | No imprecision | Unlikely | High |
| Decreased appetite | 2 | 6/763 | 1/774 | 3.25 [0.29, 36.41] | Low | No inconsistency | No indirectness | No imprecision | Unlikely | High |
| Blood creatinine increased | 1 | 3/397 | 0/400 | 7.05 [0.37, 136.10] | Low | No inconsistency | No indirectness | No imprecision | Unlikely | High |
| Severe skin reactions | 1 | 3/397 | 0/400 | 7.05 [0.37, 136.10] | Low | No inconsistency | No indirectness | No imprecision | Unlikely | High |
| Colitis | 1 | 3/397 | 0/400 | 7.05 [0.37, 136.10] | Low | No inconsistency | No indirectness | No imprecision | Unlikely | High |
| Fatigue | 3 | 6/820 | 5/803 | 1.04 [0.32, 3.37] | Low | No inconsistency | No indirectness | No imprecision | Unlikely | High |
| Constipation | 2 | 4/763 | 0/774 | 4.80 [0.55, 42.14] | Low | No inconsistency | No indirectness | No imprecision | Unlikely | High |
| Asthenia | 2 | 4/763 | 7/774 | 0.54 [0.02, 12.18] | Low | No inconsistency | No indirectness | No imprecision | Unlikely | High |
| Rash | 2 | 2/423 | 1/403 | 2.04 [0.19, 22.44] | Low | No inconsistency | No indirectness | No imprecision | Unlikely | High |
| Hepatitis | 1 | 1/397 | 0/400 | 3.02 [0.12, 73.97] | Low | No inconsistency | No indirectness | No imprecision | Unlikely | High |
| Hypophysitis | 1 | 1/397 | 0/400 | 3.02 [0.12, 73.97] | Low | No inconsistency | No indirectness | No imprecision | Unlikely | High |
| Myasthenic syndrome | 1 | 1/397 | 0/400 | 3.02 [0.12, 73.97] | Low | No inconsistency | No indirectness | No imprecision | Unlikely | High |
| Myocarditis | 1 | 1/397 | 0/400 | 3.02 [0.12, 73.97] | Low | No inconsistency | No indirectness | No imprecision | Unlikely | High |
| Myositis | 1 | 1/397 | 0/400 | 3.02 [0.12, 73.97] | Low | No inconsistency | No indirectness | No imprecision | Unlikely | High |
| Arthralgia | 2 | 1/423 | 1/403 | 1.02 [0.06, 16.28] | Low | No inconsistency | No indirectness | No imprecision | Unlikely | High |
| Pruritus | 2 | 1/423 | 0/403 | 3.07 [0.13, 75.00] | Low | No inconsistency | No indirectness | No imprecision | Unlikely | High |
| Peripheral sensory neuropathy | 1 | 0/57 | 1/29 | 0.17 [0.01, 4.11] | Low | No inconsistency | No indirectness | No imprecision | Unlikely | High |
| Myalgia | 1 | 0/57 | 0/29 | Not estimable | Low | No inconsistency | No indirectness | No imprecision | Unlikely | High |
| Oral mucositis | 1 | 0/57 | 0/29 | Not estimable | Low | No inconsistency | No indirectness | No imprecision | Unlikely | High |
| Paresthesia | 1 | 0/57 | 0/29 | Not estimable | Low | No inconsistency | No indirectness | No imprecision | Unlikely | High |
| Gastrointestinal disorders | 1 | 0/57 | 0/29 | Not estimable | Low | No inconsistency | No indirectness | No imprecision | Unlikely | High |
| Insomnia | 1 | 0/366 | 0/374 | Not estimable | Low | No inconsistency | No indirectness | No imprecision | Unlikely | High |
| Alopecia | 3 | 0/820 | 2/803 | 0.34 [0.04, 3.24] | Low | No inconsistency | No indirectness | No imprecision | Unlikely | High |
| Hypothyroidism | 2 | 0/763 | 0/774 | Not estimable | Low | No inconsistency | No indirectness | No imprecision | Unlikely | High |
| Hyperthyroidism | 1 | 0/397 | 0/400 | Not estimable | Low | No inconsistency | No indirectness | No imprecision | Unlikely | High |
| Infusion reactions | 1 | 0/397 | 0/400 | Not estimable | Low | No inconsistency | No indirectness | No imprecision | Unlikely | High |
| Thyroiditis | 1 | 0/397 | 0/400 | Not estimable | Low | No inconsistency | No indirectness | No imprecision | Unlikely | High |
| Pancreatitis | 1 | 0/397 | 0/400 | Not estimable | Low | No inconsistency | No indirectness | No imprecision | Unlikely | High |
| Uveitis | 1 | 0/397 | 0/400 | Not estimable | Low | No inconsistency | No indirectness | No imprecision | Unlikely | High |
| Vasculitis | 1 | 0/397 | 1/400 | 0.34 [0.01, 8.22] | Low | No inconsistency | No indirectness | No imprecision | Unlikely | High |
| **Grade 3-5 adverse events during the surgical treatment phase** | | | |  |  |  |  |  |  |  |
| Anemia | 1 | 16/397 | 15/400 | 1.07 [0.54, 2.14] | Low | No inconsistency | No indirectness | No imprecision | Unlikely | High |
| Pneumonia | 1 | 8/397 | 8/400 | 1.01 [0.38, 2.66] | Low | No inconsistency | No indirectness | No imprecision | Unlikely | High |
| Procedural pain | 1 | 5/397 | 2/400 | 2.52 [0.49, 12.91] | Low | No inconsistency | No indirectness | No imprecision | Unlikely | High |
| Dyspnea | 1 | 3/397 | 1/400 | 3.02 [0.32, 28.93] | Low | No inconsistency | No indirectness | No imprecision | Unlikely | High |
| Pneumothorax | 1 | 3/397 | 3/400 | 1.01 [0.20, 4.96] | Low | No inconsistency | No indirectness | No imprecision | Unlikely | High |
| Diarrhea | 1 | 2/397 | 1/400 | 2.02 [0.18, 22.13] | Low | No inconsistency | No indirectness | No imprecision | Unlikely | High |
| Incision site pain | 2 | 2/763 | 2/774 | 0.97 [0.14, 6.59] | Low | No inconsistency | No indirectness | No imprecision | Unlikely | High |
| Chest pain | 1 | 1/397 | 0/400 | 3.02 [0.12, 73.97] | Low | No inconsistency | No indirectness | No imprecision | Unlikely | High |
| Pleural effusion | 1 | 1/397 | 5/400 | 0.20 [0.02, 1.72] | Low | No inconsistency | No indirectness | No imprecision | Unlikely | High |
| Atrial fibrillation | 1 | 1/397 | 3/400 | 0.34 [0.04, 3.21] | Low | No inconsistency | No indirectness | No imprecision | Unlikely | High |
| Cough | 1 | 0/397 | 0/400 | Not estimable | Low | No inconsistency | No indirectness | No imprecision | Unlikely | High |
| Constipation | 1 | 0/397 | 1/400 | 0.34 [0.01, 8.22] | Low | No inconsistency | No indirectness | No imprecision | Unlikely | High |
| Wound complication | 1 | 0/397 | 0/400 | Not estimable | Low | No inconsistency | No indirectness | No imprecision | Unlikely | High |
| Productive cough | 1 | 0/397 | 0/400 | Not estimable | Low | No inconsistency | No indirectness | No imprecision | Unlikely | High |
| Subcutaneous emphysema | 1 | 0/397 | 0/400 | Not estimable | Low | No inconsistency | No indirectness | No imprecision | Unlikely | High |
| **Grade 3-5 adverse events during the adjuvant treatment phase** | | | |  |  |  |  |  |  |  |
| Hyperthyroidism | 1 | 7/397 | 0/400 | 15.11 [0.87, 263.73] | Low | No inconsistency | No indirectness | No imprecision | Unlikely | High |
| Pneumonitis | 1 | 3/397 | 1/400 | 3.02 [0.32, 28.93] | Low | No inconsistency | No indirectness | No imprecision | Unlikely | High |
| Severe skin reactions | 1 | 3/397 | 0/400 | 7.05 [0.37, 136.10] | Low | No inconsistency | No indirectness | No imprecision | Unlikely | High |
| Diarrhea | 2 | 3/454 | 0/429 | 7.05 [0.37, 136.10] | Low | No inconsistency | No indirectness | No imprecision | Unlikely | High |
| Pruritus | 2 | 2/454 | 0/429 | 5.04 [0.24, 104.60] | Low | No inconsistency | No indirectness | No imprecision | Unlikely | High |
| Rash | 1 | 1/397 | 0/400 | 3.02 [0.12, 73.97] | Low | No inconsistency | No indirectness | No imprecision | Unlikely | High |
| Adrenal insufficiency | 1 | 1/397 | 0/400 | 3.02 [0.12, 73.97] | Low | No inconsistency | No indirectness | No imprecision | Unlikely | High |
| Hypothyroidism | 2 | 0/454 | 0/429 | Not estimable | Low | No inconsistency | No indirectness | No imprecision | Unlikely | High |
| Colitis | 1 | 0/397 | 0/400 | Not estimable | Low | No inconsistency | No indirectness | No imprecision | Unlikely | High |
| Hepatitis | 1 | 0/397 | 2/400 | 0.20 [0.01, 4.18] | Low | No inconsistency | No indirectness | No imprecision | Unlikely | High |
| Hypophysitis | 1 | 0/397 | 0/400 | Not estimable | Low | No inconsistency | No indirectness | No imprecision | Unlikely | High |
| Guillain-Barré syndrome | 1 | 0/397 | 1/400 | 0.34 [0.01, 8.22] | Low | No inconsistency | No indirectness | No imprecision | Unlikely | High |
| Infusion reactions | 1 | 0/397 | 0/400 | Not estimable | Low | No inconsistency | No indirectness | No imprecision | Unlikely | High |
| Pancreatitis | 1 | 0/397 | 1/400 | 0.34 [0.01, 8.22] | Low | No inconsistency | No indirectness | No imprecision | Unlikely | High |
| Alanine aminotransferase increased | 1 | 0/57 | 0/29 | Not estimable | Low | No inconsistency | No indirectness | No imprecision | Unlikely | High |
| Anemia | 1 | 0/57 | 0/29 | Not estimable | Low | No inconsistency | No indirectness | No imprecision | Unlikely | High |
| Arthralgia | 1 | 0/57 | 0/29 | Not estimable | Low | No inconsistency | No indirectness | No imprecision | Unlikely | High |
| Fatigue | 1 | 0/57 | 0/29 | Not estimable | Low | No inconsistency | No indirectness | No imprecision | Unlikely | High |
| Myalgia | 1 | 0/57 | 0/29 | Not estimable | Low | No inconsistency | No indirectness | No imprecision | Unlikely | High |
| Peripheral sensory neuropathy | 1 | 0/57 | 0/29 | Not estimable | Low | No inconsistency | No indirectness | No imprecision | Unlikely | High |

**Abbreviations:** CI: confidence interval; Event-free survival; EFSR: Event-free survival rate; GRADE: Grading of Recommendations, Assessment, Development, and Evaluation; OS: Overall survival; OSR: Overall survival rate; RR: Risk ratio; HR: Hazard ratio.

^a^ Differences: HR for OS and EFS; RR for OSR, EFSR, pathological responses, and adverse events.

^b^ Risk of bias assessed using the Jadad scale for randomized controlled trials.

^c^ Publication bias was explored through visual inspection of the funnel plots.
