# Supplementary material for: Perioperative immunotherapy for stage II-III non-small cell lung cancer: a meta-analysis base on randomized controlled trials
Source: Front Oncol. 2024 Feb 22;14:1351359. doi: 10.3389/fonc.2024.1351359 (PMC10917905; doi:10.3389/fonc.2024.1351359)
Supplement: Supplementary file 15 [file Table_5.doc]

**Table S5** Total adverse events during the neoadjuvant treatment phase.

| **Adverse events** | **Studies involved** | **PIO** | | **PP** | | **Risk ratio [95% CI]** | **P** |
| --- | --- | --- | --- | --- | --- | --- | --- |
|  |  | **Event/total** | **%** | **Event/total** | **%** |  |  |
| Nausea | 2 | 314/763 | 41.15% | 317/774 | 40.96% | 1.00 [0.89, 1.13] | 0.96 |
| Anemia | 2 | 276/763 | 36.17% | 260/774 | 33.59% | 1.08 [0.94, 1.23] | 0.29 |
| Neutrophil count decreased | 2 | 231/763 | 30.28% | 223/774 | 28.81% | 1.05 [0.90, 1.22] | 0.54 |
| Constipation | 2 | 205/763 | 26.87% | 183/774 | 23.64% | 1.14 [0.96, 1.35] | 0.15 |
| Fatigue | 3 | 190/820 | 23.17% | 150/803 | 18.68% | 1.18 [0.98, 1.43] | 0.08 |
| Peripheral sensory neuropathy | 1 | 13/57 | 22.81% | 10/29 | 34.48% | 0.66 [0.33, 1.32] | 0.24 |
| Decreased appetite | 2 | 159/763 | 20.84% | 156/774 | 20.16% | 1.03 [0.85, 1.26] | 0.74 |
| Myalgia | 1 | 11/57 | 19.30% | 3/29 | 10.34% | 1.87 [0.56, 6.17] | 0.31 |
| Neutropenia | 1 | 68/366 | 18.58% | 71/374 | 18.98% | 0.98 [0.73, 1.32] | 0.89 |
| White blood cell count decreased | 2 | 136/763 | 17.82% | 130/774 | 16.80% | 1.06 [0.86, 1.31] | 0.61 |
| Vomiting | 2 | 119/763 | 15.60% | 99/774 | 12.79% | 1.22 [0.95, 1.56] | 0.12 |
| Rash | 2 | 63/423 | 14.89% | 34/403 | 8.44% | 1.80 [1.21, 2.68] | 0.004 |
| Alopecia | 3 | 122/820 | 14.88% | 113/803 | 14.07% | 1.02 [0.80, 1.29] | 0.89 |
| Blood creatinine increased | 1 | 53/397 | 13.35% | 44/400 | 11.00% | 1.21 [0.83, 1.77] | 0.31 |
| Platelet count decreased | 2 | 101/763 | 13.24% | 105/774 | 13.57% | 0.97 [0.76, 1.25] | 0.83 |
| Pruritus | 2 | 54/423 | 12.77% | 24/403 | 5.96% | 2.14 [1.35, 3.40] | 0.001 |
| Asthenia | 2 | 93/763 | 12.19% | 109/774 | 14.08% | 0.87 [0.67, 1.12] | 0.27 |
| Diarrhea | 3 | 99/820 | 12.07% | 98/803 | 12.20% | 0.99 [0.76, 1.29] | 0.93 |
| Insomnia | 1 | 41/366 | 11.20% | 46/374 | 12.30% | 0.91 [0.61, 1.35] | 0.64 |
| Alanine aminotransferase increased | 2 | 74/763 | 9.70% | 44/774 | 5.68% | 1.70 [1.19, 2.44] | 0.004 |
| Leukopenia | 1 | 34/366 | 9.29% | 30/374 | 8.02% | 1.16 [0.72, 1.85] | 0.54 |
| Arthralgia | 2 | 38/423 | 8.98% | 28/403 | 6.95% | 1.08 [0.68, 1.70] | 0.76 |
| Hypothyroidism | 2 | 67/763 | 8.78% | 11/774 | 1.42% | 6.18 [3.30, 11.61] | <0.00001 |
| Febrile neutropenia | 1 | 5/57 | 8.77% | 0/29 | 0.00% | 5.69 [0.33, 99.48] | 0.23 |
| Thrombocytopenia | 1 | 25/366 | 6.83% | 29/374 | 7.75% | 0.88 [0.53, 1.47] | 0.63 |
| Hyperthyroidism | 1 | 15/397 | 3.78% | 6/400 | 1.50% | 2.52 [0.99, 6.43] | 0.05 |
| Pneumonitis | 1 | 11/397 | 2.77% | 3/400 | 0.75% | 3.69 [1.04, 13.14] | 0.04 |
| Gastrointestinal disorders | 1 | 1/57 | 1.75% | 3/29 | 10.34% | 0.17 [0.02, 1.56] | 0.12 |
| Infusion reactions | 1 | 5/397 | 1.26% | 4/400 | 1.00% | 1.26 [0.34, 4.66] | 0.73 |
| Severe skin reactions | 1 | 5/397 | 1.26% | 0/400 | 0.00% | 11.08 [0.61, 199.76] | 0.10 |
| Colitis | 1 | 3/397 | 0.76% | 0/400 | 0.00% | 7.05 [0.37, 136.10] | 0.20 |
| Thyroiditis | 1 | 3/397 | 0.76% | 1/400 | 0.25% | 3.02 [0.32, 28.93] | 0.34 |
| Hepatitis | 1 | 1/397 | 0.25% | 0/400 | 0.00% | 3.02 [0.12, 73.97] | 0.50 |
| Hypophysitis | 1 | 1/397 | 0.25% | 0/400 | 0.00% | 3.02 [0.12, 73.97] | 0.50 |
| Myasthenic syndrome | 1 | 1/397 | 0.25% | 0/400 | 0.00% | 3.02 [0.12, 73.97] | 0.50 |
| Myocarditis | 1 | 1/397 | 0.25% | 0/400 | 0.00% | 3.02 [0.12, 73.97] | 0.50 |
| Myositis | 1 | 1/397 | 0.25% | 0/400 | 0.00% | 3.02 [0.12, 73.97] | 0.50 |
| Oral mucositis | 1 | 0/57 | 0.00% | 3/29 | 10.34% | 0.07 [0.00, 1.38] | 0.08 |
| Paresthesia | 1 | 0/57 | 0.00% | 3/29 | 10.34% | 0.07 [0.00, 1.38] | 0.08 |
| Pancreatitis | 1 | 0/397 | 0.00% | 1/400 | 0.25% | 0.34 [0.01, 8.22] | 0.50 |
| Uveitis | 1 | 0/397 | 0.00% | 1/400 | 0.25% | 0.34 [0.01, 8.22] | 0.50 |
| Vasculitis | 1 | 0/397 | 0.00% | 2/400 | 0.50% | 0.20 [0.01, 4.18] | 0.30 |

**Abbreviations:** CI: confidence interval; P: Probability; PIO: Perioperative immunotherapy; PP: Perioperative placebo.
