# Supplementary material for: Perioperative immunotherapy for stage II-III non-small cell lung cancer: a meta-analysis base on randomized controlled trials
Source: Front Oncol. 2024 Feb 22;14:1351359. doi: 10.3389/fonc.2024.1351359 (PMC10917905; doi:10.3389/fonc.2024.1351359)
Supplement: Supplementary file 16 [file Table_6.doc]

**Table S6** Grade 3-5 adverse events during the neoadjuvant treatment phase.

| **Adverse events** | **Studies involved** | **PIO** | | **PP** | | **Risk ratio [95% CI]** | **P** |
| --- | --- | --- | --- | --- | --- | --- | --- |
|  |  | **Event/total** | **%** | **Event/total** | **%** |  |  |
| Neutrophil count decreased | 2 | 121/763 | 15.86% | 121/774 | 15.63% | 1.02 [0.81, 1.28] | 0.89 |
| Neutropenia | 1 | 36/366 | 9.84% | 38/374 | 10.16% | 0.97 [0.63, 1.49] | 0.88 |
| Anemia | 2 | 55/763 | 7.21% | 48/774 | 6.20% | 1.16 [0.80, 1.69] | 0.43 |
| Febrile neutropenia | 1 | 4/57 | 7.02% | 0/29 | 0.00% | 4.66 [0.26, 83.62] | 0.30 |
| White blood cell count decreased | 2 | 29/763 | 3.80% | 34/774 | 4.39% | 0.87 [0.53, 1.41] | 0.56 |
| Platelet count decreased | 2 | 27/763 | 3.54% | 36/774 | 4.65% | 0.76 [0.47, 1.25] | 0.28 |
| Leukopenia | 1 | 9/366 | 2.46% | 12/374 | 3.21% | 0.77 [0.33, 1.80] | 0.54 |
| Thrombocytopenia | 1 | 6/366 | 1.64% | 9/374 | 2.41% | 0.68 [0.24, 1.89] | 0.46 |
| Pneumonitis | 1 | 5/397 | 1.26% | 0/400 | 0.00% | 11.08 [0.61, 199.76] | 0.10 |
| Nausea | 2 | 8/763 | 1.05% | 7/774 | 0.90% | 1.15 [0.42, 3.16] | 0.78 |
| Diarrhea | 3 | 8/820 | 0.98% | 6/803 | 0.75% | 1.14 [0.40, 3.27] | 0.81 |
| Vomiting | 2 | 7/763 | 0.92% | 5/774 | 0.65% | 1.44 [0.29, 7.08] | 0.65 |
| Alanine aminotransferase increased | 2 | 7/763 | 0.92% | 3/774 | 0.39% | 2.36 [0.61, 9.09] | 0.21 |
| Decreased appetite | 2 | 6/763 | 0.79% | 1/774 | 0.13% | 3.25 [0.29, 36.41] | 0.34 |
| Blood creatinine increased | 1 | 3/397 | 0.76% | 0/400 | 0.00% | 7.05 [0.37, 136.10] | 0.20 |
| Severe skin reactions | 1 | 3/397 | 0.76% | 0/400 | 0.00% | 7.05 [0.37, 136.10] | 0.20 |
| Colitis | 1 | 3/397 | 0.76% | 0/400 | 0.00% | 7.05 [0.37, 136.10] | 0.20 |
| Fatigue | 3 | 6/820 | 0.73% | 5/803 | 0.62% | 1.04 [0.32, 3.37] | 0.94 |
| Constipation | 2 | 4/763 | 0.52% | 0/774 | 0.00% | 4.80 [0.55, 42.14] | 0.16 |
| Asthenia | 2 | 4/763 | 0.52% | 7/774 | 0.90% | 0.54 [0.02, 12.18] | 0.69 |
| Rash | 2 | 2/423 | 0.47% | 1/403 | 0.25% | 2.04 [0.19, 22.44] | 0.56 |
| Hepatitis | 1 | 1/397 | 0.25% | 0/400 | 0.00% | 3.02 [0.12, 73.97] | 0.50 |
| Hypophysitis | 1 | 1/397 | 0.25% | 0/400 | 0.00% | 3.02 [0.12, 73.97] | 0.50 |
| Myasthenic syndrome | 1 | 1/397 | 0.25% | 0/400 | 0.00% | 3.02 [0.12, 73.97] | 0.50 |
| Myocarditis | 1 | 1/397 | 0.25% | 0/400 | 0.00% | 3.02 [0.12, 73.97] | 0.50 |
| Myositis | 1 | 1/397 | 0.25% | 0/400 | 0.00% | 3.02 [0.12, 73.97] | 0.50 |
| Arthralgia | 2 | 1/423 | 0.24% | 1/403 | 0.25% | 1.02 [0.06, 16.28] | 0.99 |
| Pruritus | 2 | 1/423 | 0.24% | 0/403 | 0.00% | 3.07 [0.13, 75.00] | 0.49 |
| Peripheral sensory neuropathy | 1 | 0/57 | 0.00% | 1/29 | 3.45% | 0.17 [0.01, 4.11] | 0.28 |
| Myalgia | 1 | 0/57 | 0.00% | 0/29 | 0.00% | Not estimable | - |
| Oral mucositis | 1 | 0/57 | 0.00% | 0/29 | 0.00% | Not estimable | - |
| Paresthesia | 1 | 0/57 | 0.00% | 0/29 | 0.00% | Not estimable | - |
| Gastrointestinal disorders | 1 | 0/57 | 0.00% | 0/29 | 0.00% | Not estimable | - |
| Insomnia | 1 | 0/366 | 0.00% | 0/374 | 0.00% | Not estimable | - |
| Alopecia | 3 | 0/820 | 0.00% | 2/803 | 0.25% | 0.34 [0.04, 3.24] | 0.35 |
| Hypothyroidism | 2 | 0/763 | 0.00% | 0/774 | 0.00% | Not estimable | - |
| Hyperthyroidism | 1 | 0/397 | 0.00% | 0/400 | 0.00% | Not estimable | - |
| Infusion reactions | 1 | 0/397 | 0.00% | 0/400 | 0.00% | Not estimable | - |
| Thyroiditis | 1 | 0/397 | 0.00% | 0/400 | 0.00% | Not estimable | - |
| Pancreatitis | 1 | 0/397 | 0.00% | 0/400 | 0.00% | Not estimable | - |
| Uveitis | 1 | 0/397 | 0.00% | 0/400 | 0.00% | Not estimable | - |
| Vasculitis | 1 | 0/397 | 0.00% | 1/400 | 0.25% | 0.34 [0.01, 8.22] | 0.50 |

**Abbreviations:** CI: confidence interval; P: Probability; PIO: Perioperative immunotherapy; PP: Perioperative placebo.
