# Supplementary material for: Perioperative immunotherapy for stage II-III non-small cell lung cancer: a meta-analysis base on randomized controlled trials
Source: Front Oncol. 2024 Feb 22;14:1351359. doi: 10.3389/fonc.2024.1351359 (PMC10917905; doi:10.3389/fonc.2024.1351359)
Supplement: Supplementary file 17 [file Table_7.doc]

**Table S7** Total adverse events during the surgical treatment phase.

| **Adverse events** | **Studies involved** | **PIO** | | **PP** | | **Risk ratio [95% CI]** | **P** |
| --- | --- | --- | --- | --- | --- | --- | --- |
|  |  | **Event/total** | **%** | **Event/total** | **%** |  |  |
| Procedural pain | 1 | 58/397 | 14.61% | 58/400 | 14.50% | 1.01 [0.72, 1.41] | 0.97 |
| Anemia | 1 | 48/397 | 12.09% | 51/400 | 12.75% | 0.95 [0.66, 1.37] | 0.78 |
| Incision site pain | 2 | 64/763 | 8.39% | 62/774 | 8.01% | 1.06 [0.72, 1.55] | 0.76 |
| Dyspnea | 1 | 33/397 | 8.31% | 20/400 | 5.00% | 1.66 [0.97, 2.85] | 0.06 |
| Cough | 1 | 32/397 | 8.06% | 27/400 | 6.75% | 1.19 [0.73, 1.96] | 0.48 |
| Pneumothorax | 2 | 28/454 | 6.17% | 27/429 | 6.29% | 1.09 [0.38, 3.18] | 0.87 |
| Constipation | 1 | 21/397 | 5.29% | 28/400 | 7.00% | 0.76 [0.44, 1.31] | 0.32 |
| Air Leakage | 1 | 3/57 | 5.26% | 2/29 | 6.90% | 0.76 [0.13, 4.32] | 0.76 |
| Chest pain | 1 | 19/397 | 4.79% | 12/400 | 3.00% | 1.60 [0.78, 3.24] | 0.20 |
| Wound complication | 1 | 18/397 | 4.53% | 18/400 | 4.50% | 1.01 [0.53, 1.91] | 0.98 |
| Pleural effusion | 2 | 20/454 | 4.41% | 20/429 | 4.66% | 1.09 [0.29, 4.07] | 0.90 |
| Diarrhea | 1 | 17/397 | 4.28% | 4/400 | 1.00% | 4.28 [1.45, 12.61] | 0.008 |
| Pneumonia | 1 | 14/397 | 3.53% | 20/400 | 5.00% | 0.71 [0.36, 1.38] | 0.31 |
| Respiratory Insufficiency | 1 | 2/57 | 3.51% | 0/29 | 0.00% | 2.59 [0.13, 52.16] | 0.54 |
| Productive cough | 1 | 12/397 | 3.02% | 18/400 | 4.50% | 0.67 [0.33, 1.38] | 0.28 |
| Subcutaneous emphysema | 2 | 9/454 | 1.98% | 18/429 | 4.20% | 0.48 [0.22, 1.07] | 0.07 |
| Chylothorax | 1 | 1/57 | 1.75% | 0/29 | 0.00% | 1.55 [0.07, 36.95] | 0.79 |
| Arrhythmia | 1 | 1/57 | 1.75% | 2/29 | 6.90% | 0.25 [0.02, 2.69] | 0.26 |
| Atelectasis | 1 | 1/57 | 1.75% | 1/29 | 3.45% | 0.51 [0.03, 7.84] | 0.63 |
| Pulmonary Thromboembolism | 1 | 1/57 | 1.75% | 0/29 | 0.00% | 1.55 [0.07, 36.95] | 0.79 |
| Atrial fibrillation | 1 | 6/397 | 1.51% | 17/400 | 4.25% | 0.36 [0.14, 0.89] | 0.03 |

**Abbreviations:** CI: confidence interval; P: Probability; PIO: Perioperative immunotherapy; PP: Perioperative placebo.
