# Supplementary material for: Perioperative immunotherapy for stage II-III non-small cell lung cancer: a meta-analysis base on randomized controlled trials
Source: Front Oncol. 2024 Feb 22;14:1351359. doi: 10.3389/fonc.2024.1351359 (PMC10917905; doi:10.3389/fonc.2024.1351359)
Supplement: Supplementary file 18 [file Table_8.doc]

**Table S8** Grade 3-5 adverse events during the surgical treatment phase.

| **Adverse events** | **Studies involved** | **PIO** | | **PP** | | **Risk ratio [95% CI]** | **P** |
| --- | --- | --- | --- | --- | --- | --- | --- |
|  |  | **Event/total** | **%** | **Event/total** | **%** |  |  |
| Anemia | 1 | 16/397 | 4.03% | 15/400 | 3.75% | 1.07 [0.54, 2.14] | 0.84 |
| Pneumonia | 1 | 8/397 | 2.02% | 8/400 | 2.00% | 1.01 [0.38, 2.66] | 0.99 |
| Procedural pain | 1 | 5/397 | 1.26% | 2/400 | 0.50% | 2.52 [0.49, 12.91] | 0.27 |
| Dyspnea | 1 | 3/397 | 0.76% | 1/400 | 0.25% | 3.02 [0.32, 28.93] | 0.34 |
| Pneumothorax | 1 | 3/397 | 0.76% | 3/400 | 0.75% | 1.01 [0.20, 4.96] | 0.99 |
| Diarrhea | 1 | 2/397 | 0.50% | 1/400 | 0.25% | 2.02 [0.18, 22.13] | 0.57 |
| Incision site pain | 2 | 2/763 | 0.26% | 2/774 | 0.26% | 0.97 [0.14, 6.59] | 0.97 |
| Chest pain | 1 | 1/397 | 0.25% | 0/400 | 0.00% | 3.02 [0.12, 73.97] | 0.50 |
| Pleural effusion | 1 | 1/397 | 0.25% | 5/400 | 1.25% | 0.20 [0.02, 1.72] | 0.14 |
| Atrial fibrillation | 1 | 1/397 | 0.25% | 3/400 | 0.75% | 0.34 [0.04, 3.21] | 0.34 |
| Cough | 1 | 0/397 | 0.00% | 0/400 | 0.00% | Not estimable | - |
| Constipation | 1 | 0/397 | 0.00% | 1/400 | 0.25% | 0.34 [0.01, 8.22] | 0.50 |
| Wound complication | 1 | 0/397 | 0.00% | 0/400 | 0.00% | Not estimable | - |
| Productive cough | 1 | 0/397 | 0.00% | 0/400 | 0.00% | Not estimable | - |
| Subcutaneous emphysema | 1 | 0/397 | 0.00% | 0/400 | 0.00% | Not estimable | - |

**Abbreviations:** CI: confidence interval; P: Probability; PIO: Perioperative immunotherapy; PP: Perioperative placebo.
