# Supplementary material for: Perioperative immunotherapy for stage II-III non-small cell lung cancer: a meta-analysis base on randomized controlled trials
Source: Front Oncol. 2024 Feb 22;14:1351359. doi: 10.3389/fonc.2024.1351359 (PMC10917905; doi:10.3389/fonc.2024.1351359)
Supplement: Supplementary file 19 [file Table_9.doc]

**Table S9** Total adverse events during the adjuvant treatment phase.

| **Adverse events** | **Studies involved** | **PIO** | | **PP** | | **Risk ratio [95% CI]** | **P** |
| --- | --- | --- | --- | --- | --- | --- | --- |
|  |  | **Event/total** | **%** | **Event/total** | **%** |  |  |
| Fatigue | 1 | 9/57 | 15.79% | 0/29 | 0.00% | 9.83 [0.59, 163.15] | 0.11 |
| Pruritus | 2 | 28/454 | 6.17% | 6/429 | 1.40% | 4.09 [1.75, 9.55] | 0.001 |
| Arthralgia | 1 | 3/57 | 5.26% | 0/29 | 0.00% | 3.62 [0.19, 67.82] | 0.39 |
| Rash | 1 | 18/397 | 4.53% | 8/400 | 2.00% | 2.27 [1.00, 5.15] | 0.05 |
| Hypothyroidism | 2 | 20/454 | 4.41% | 2/429 | 0.47% | 7.46 [2.07, 26.89] | 0.002 |
| Diarrhea | 2 | 18/454 | 3.96% | 12/429 | 2.80% | 1.38 [0.67, 2.84] | 0.38 |
| Alanine aminotransferase increased | 1 | 2/57 | 3.51% | 0/29 | 0.00% | 2.59 [0.13, 52.16] | 0.54 |
| Anemia | 1 | 2/57 | 3.51% | 0/29 | 0.00% | 2.59 [0.13, 52.16] | 0.54 |
| Myalgia | 1 | 2/57 | 3.51% | 0/29 | 0.00% | 2.59 [0.13, 52.16] | 0.54 |
| Peripheral sensory neuropathy | 1 | 2/57 | 3.51% | 0/29 | 0.00% | 2.59 [0.13, 52.16] | 0.54 |
| Pneumonitis | 1 | 11/397 | 2.77% | 4/400 | 1.00% | 2.77 [0.89, 8.63] | 0.08 |
| Hyperthyroidism | 1 | 8/397 | 2.02% | 0/400 | 0.00% | 17.13 [0.99, 295.75] | 0.05 |
| Severe skin reactions | 1 | 4/397 | 1.01% | 0/400 | 0.00% | 9.07 [0.49, 167.87] | 0.14 |
| Colitis | 1 | 2/397 | 0.50% | 0/400 | 0.00% | 5.04 [0.24, 104.60] | 0.30 |
| Hepatitis | 1 | 2/397 | 0.50% | 2/400 | 0.50% | 1.01 [0.14, 7.12] | 0.99 |
| Adrenal insufficiency | 1 | 1/397 | 0.25% | 0/400 | 0.00% | 3.02 [0.12, 73.97] | 0.50 |
| Hypophysitis | 1 | 1/397 | 0.25% | 0/400 | 0.00% | 3.02 [0.12, 73.97] | 0.50 |
| Guillain-Barré syndrome | 1 | 0/397 | 0.00% | 1/400 | 0.25% | 0.34 [0.01, 8.22] | 0.50 |
| Infusion reactions | 1 | 0/397 | 0.00% | 2/400 | 0.50% | 0.20 [0.01, 4.18] | 0.30 |
| Pancreatitis | 1 | 0/397 | 0.00% | 1/400 | 0.25% | 0.34 [0.01, 8.22] | 0.50 |

**Abbreviations:** CI: confidence interval; P: Probability; PIO: Perioperative immunotherapy; PP: Perioperative placebo.
