# Supplementary material for: Perioperative immunotherapy for stage II-III non-small cell lung cancer: a meta-analysis base on randomized controlled trials
Source: Front Oncol. 2024 Feb 22;14:1351359. doi: 10.3389/fonc.2024.1351359 (PMC10917905; doi:10.3389/fonc.2024.1351359)
Supplement: Supplementary file 20 [file Table_10.doc]

**Table S10** Grade 3-5 adverse events during the adjuvant treatment phase.

| **Adverse events** | **Studies involved** | **PIO** | | **PP** | | **Risk ratio [95% CI]** | **P** |
| --- | --- | --- | --- | --- | --- | --- | --- |
|  |  | **Event/total** | **%** | **Event/total** | **%** |  |  |
| Hyperthyroidism | 1 | 7/397 | 1.76% | 0/400 | 0.00% | 15.11 [0.87, 263.73] | 0.06 |
| Pneumonitis | 1 | 3/397 | 0.76% | 1/400 | 0.25% | 3.02 [0.32, 28.93] | 0.34 |
| Severe skin reactions | 1 | 3/397 | 0.76% | 0/400 | 0.00% | 7.05 [0.37, 136.10] | 0.20 |
| Diarrhea | 2 | 3/454 | 0.66% | 0/429 | 0.00% | 7.05 [0.37, 136.10] | 0.20 |
| Pruritus | 2 | 2/454 | 0.44% | 0/429 | 0.00% | 5.04 [0.24, 104.60] | 0.30 |
| Rash | 1 | 1/397 | 0.25% | 0/400 | 0.00% | 3.02 [0.12, 73.97] | 0.50 |
| Adrenal insufficiency | 1 | 1/397 | 0.25% | 0/400 | 0.00% | 3.02 [0.12, 73.97] | 0.50 |
| Hypothyroidism | 2 | 0/454 | 0.00% | 0/429 | 0.00% | Not estimable | - |
| Colitis | 1 | 0/397 | 0.00% | 0/400 | 0.00% | Not estimable | - |
| Hepatitis | 1 | 0/397 | 0.00% | 2/400 | 0.50% | 0.20 [0.01, 4.18] | 0.30 |
| Hypophysitis | 1 | 0/397 | 0.00% | 0/400 | 0.00% | Not estimable | - |
| Guillain-Barré syndrome | 1 | 0/397 | 0.00% | 1/400 | 0.25% | 0.34 [0.01, 8.22] | 0.50 |
| Infusion reactions | 1 | 0/397 | 0.00% | 0/400 | 0.00% | Not estimable | - |
| Pancreatitis | 1 | 0/397 | 0.00% | 1/400 | 0.25% | 0.34 [0.01, 8.22] | 0.50 |
| Alanine aminotransferase increased | 1 | 0/57 | 0.00% | 0/29 | 0.00% | Not estimable | - |
| Anemia | 1 | 0/57 | 0.00% | 0/29 | 0.00% | Not estimable | - |
| Arthralgia | 1 | 0/57 | 0.00% | 0/29 | 0.00% | Not estimable | - |
| Fatigue | 1 | 0/57 | 0.00% | 0/29 | 0.00% | Not estimable | - |
| Myalgia | 1 | 0/57 | 0.00% | 0/29 | 0.00% | Not estimable | - |
| Peripheral sensory neuropathy | 1 | 0/57 | 0.00% | 0/29 | 0.00% | Not estimable | - |

**Abbreviations:** CI: confidence interval; P: Probability; PIO: Perioperative immunotherapy; PP: Perioperative placebo.
